# Supplementary material for: Identification and characterization of recent retrovirus in Rhinolophus ferrumequinum bats
Source: Microbiol Spectr. 2024 Apr 30;12(6):e04323-23. doi: 10.1128/spectrum.04323-23 (PMC11237596; doi:10.1128/spectrum.04323-23)
Supplement: Supplemental Figure 1 — Regression analysis. [file spectrum.04323-23-s0001.pdf]

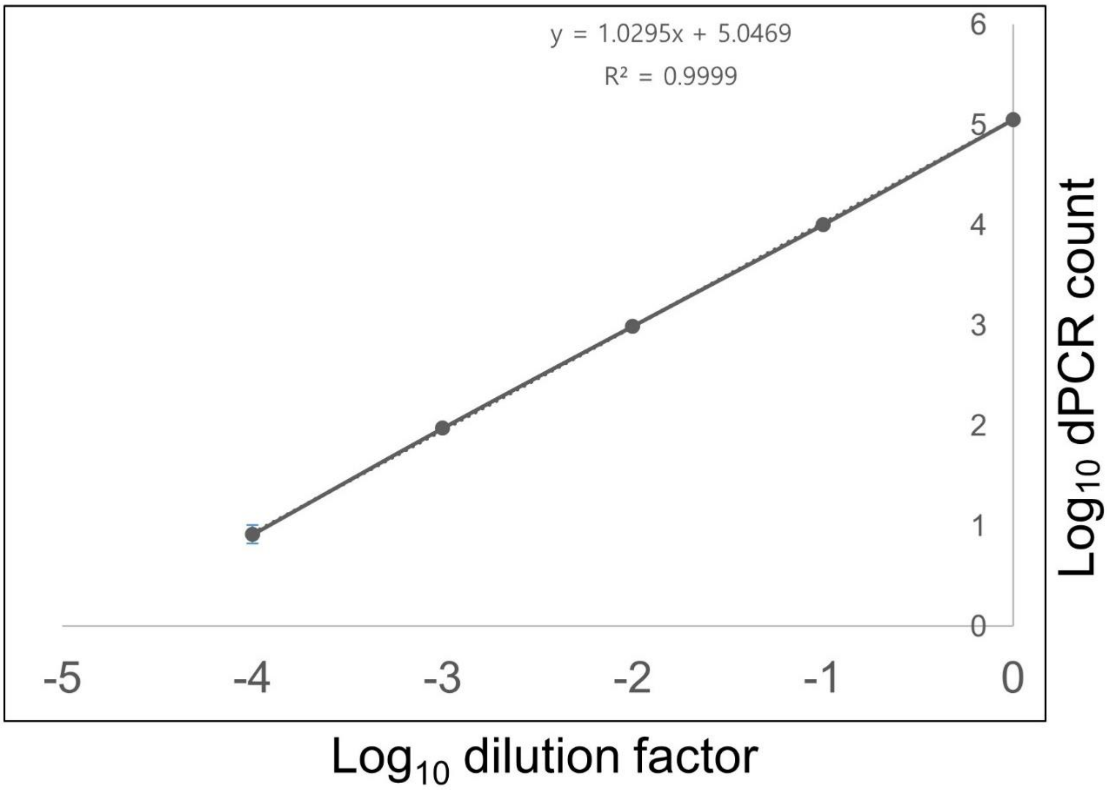

Supplementary Figure 1. Regression analysis between log10-transformed bat endogenous retrovirus counts derived from dPCR and log10-transformed the 10-fold serial dilution factors in the dPCR primer working efficacy test (R^2=0.9999). The scale blue bar represents the standard deviation.
