# Supplementary material for: Identification and characterization of recent retrovirus in Rhinolophus ferrumequinum bats
Source: Microbiol Spectr. 2024 Apr 30;12(6):e04323-23. doi: 10.1128/spectrum.04323-23 (PMC11237596; doi:10.1128/spectrum.04323-23)
Supplement: Supplemental Figure 2 — Presents heatmaps. [file spectrum.04323-23-s0002.pdf]

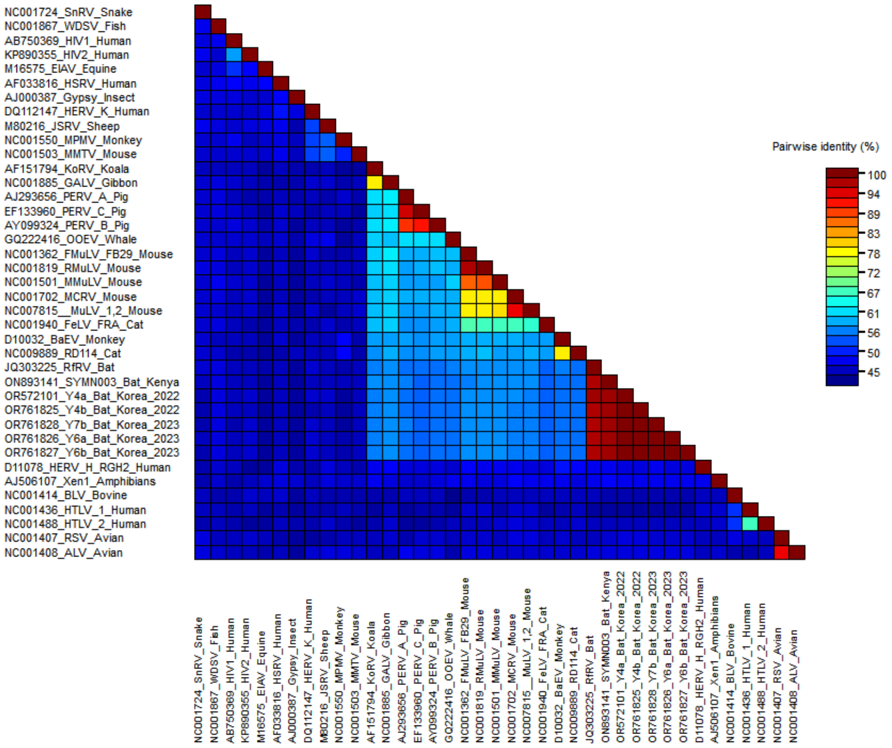

Supplementary Figure 2. Presents heatmaps illustrating whole-genome comparisons between bat retrovirus Korea strains and other retrovirus genera. The color scale represents the sequence identity percentage for each sequence pair. It is evident that the similarities between the bat retrovirus Korea strains and other strains were notably low.
