# Supplementary material for: Identification and characterization of recent retrovirus in Rhinolophus ferrumequinum bats
Source: Microbiol Spectr. 2024 Apr 30;12(6):e04323-23. doi: 10.1128/spectrum.04323-23 (PMC11237596; doi:10.1128/spectrum.04323-23)
Supplement: Supplemental material — This study sequences. [file spectrum.04323-23-s0003.pdf]

LOCUS OR572101 8363 bp RNA linear VRL 17-SEP-2023

DEFINITION bat.

ACCESSION OR572101

VERSION OR572101

KEYWORDS .

SOURCE Rhinolophus ferrumequinum retrovirus (RfRV)

ORGANISM Rhinolophus ferrumequinum retrovirus

Viruses; Riboviria; Pararnavirae; Artverviricota; Revtraviricetes;

Ortervirales; Retroviridae; Orthoretrovirinae; Gammaretrovirus; Bat

gammaretrovirus.

REFERENCE 1 (bases 1 to 8363)

AUTHORS Chung,H., Nguyen,V., Hwang,S., Chung,C. and Lee,J.

TITLE Identification of Rhinolophus ferrumequinum retrovirus of bats in

Korea

JOURNAL Unpublished

REFERENCE 2 (bases 1 to 8363)

AUTHORS Chung,H., Nguyen,V., Hwang,S., Chung,C. and Lee,J.

TITLE Direct Submission

JOURNAL Submitted (17-SEP-2023) Department of Microbiology and Immunology,

Institute for Immunology and Immunological Diseases, Yonsei

University College of Medicine, Seoul 03722 Korea., Institute for

Immunology and Immunological Diseases, Yonsei University College of

Medicine, 50-1, Yonsei-ro, Seodaemun-gu,Seoul, Korea, Seoul, Seoul

Ks031, Seoul

COMMENT ##Assembly-Data-START##

Assembly Method :: BioEdit v. 7.71

Sequencing Technology :: Illumina; Sanger dideoxy sequencing

##Assembly-Data-END##

FEATURES Location/Qualifiers

source 1..8363

/organism="Rhinolophus ferrumequinum retrovirus"

/mol_type="genomic RNA"

/isolate="Y4a"

/host="Rhinolophus ferrumequinum; bat"

/db_xref="taxon:1147756"

/country="South Korea"

/collection_date="2022-08-17"

gene 621..2348

/gene="gag protein"

CDS 621..2348

/gene="gag protein"

/codon_start=1

/product="gag protein"

/translation="MGQVQVTPKTLLLNHFPEIRAKARNHGVEVKKGKFDTFCSAEWP

TFNVGWPPQGTFPLDIIKKVRDIINRRHPDQYPYILIWQALVESPPSWLKPFIPDKPE

GPPLPLKVLTVSGPSRQPAVPTAGPKPPEKPTQGPILQEGSDKYPSLIDLDLEETPPP

YAPVAPLQPRRAPSPVAPLLPEAAAPPELPHSTASPMAPPPPGSPAPAQGPARGLKPR

RRREETPEEEPSSSTSAGAPILPVRALGGTGPDGERAYQYWPFSSSDLYNWKAQNPPF

SEDPKGLTNLFESVMHTHSPTWDDCQQLLKTLFTTEERERILTEARKNVPGDNGRPTT

LPNLIDERFPLNRLDWDFGNAEGRERLRVYRQTLMAGLRAAARRPTNLAKVKAIMQGE

NESPAVFLERLYDAYRQYTPLDPLAEENQSAVIMSFINQAAPDIRKKLYKQEGLGEMS

IRDLMKVAERVFNTRETPEKREDRIRKENQELQERIRKEDREHQSRENRRQQREMAKI

LLAGVQSTVRVGPSPAGPARPWRPRPRLDRGQCANCKEYGHWKRECPKRQGQTGQDAR

VLLAGMESD"

gene 2349..4934

/gene="pol protein"

/note="pol protein"

CDS 2349..4934

/gene="pol protein"

/codon_start=1

/product="pol protein"

/translation="GRRDSDPLPESWVTAYVEGKPVGFMVDTGAQYSVLNKPTEPLSQ

KTSLVQGATGSKAYRWTSKRQVDLGRHQVTHSFLVIPECPAPLLGRDLLTKIRAQIHF

EPDGIKLLDGQGQPLHILTLSLVDEHRLFALQDNPYNPPSTEWPRDMDYWLKTYPQAW

AEIAGVGRAARRAPVVVELKASARPIRIRQYPMSAEARKGIAPHINRLLEAGILKPCH

SAWNTPLLPVKKPGGKDYRPVQDLREVNKRVEDIHPTVPNPYTLLSHLPPSHVWYTTL

DLKDAFFSIALAPSSQHIFAFEWNNGNTGTPGQLTWTRLPQGFKNSPTLFNEALNQDL

DSFRQSHNSVTLLQYVDDLLLAAPSEAECRQATGDLLQELGQLGYRASAKKAQICRQT

VTYLGYKLKEGTRWLTEAMKETILRLPVPTSPQEVREFLGTTGYCRLWILGYAEIAKP

LYEATKDKVPWAWGSDQQKAYDELKVALLRAPALALPDPLKPFTLFVDERRGIAKGVL

MQRLGPWKRPVAYLSKKLDPVAAGWPPCLRIIAAVALMVKDADKLTFGQHLKVVTPHA

IEGVLKYPPGRWMTNARLTHYQGLLLDAPRIIFAEPTALNPATLLPTPDLRAPLHDCQ

EIMAEVTQVRPDLQDTALPNSELVWYTDGSSFVIDGVRRAGAAVVDQGGNIIWNASLS

PGTSAQKAELIALAEALERAKGRRVTVYTDSRYAFGTVHVHGAIYRERGFVTAEGKAL

RNLPEVRRLLMAVQMPRAVAVVHIPGHQSAQTPEAEGNRRADEAAKAVAVASSALALT

LPTPELPRLPPRPDYTPEDLRWIQNHHCPESDQQGWHRDTERRLILPAQLDCFFSPTC

IKPPT"

gene 5990..7867

/gene="envelope glycoprotein"

CDS 5990..7867

/gene="envelope glycoprotein"

/codon_start=1

/product="envelope glycoprotein"

/translation="MEWLLALTLLNIWEKSHAGINPHQPHKLTWTLTDGQTQTTLNST

THTAPINTWWPDLFFDLRDIFGTKRGRQYDYSVRSKRAVIDTSQGHSAQGFWACPGNL

RNNWKTCGGPDRYYWGSWSWVTSYDGPRQWDVGNRDLVKFSFRDPHNRVPQVCVQFNQ

DVARRERGWLSGLTWGFQLDIGRWAWIGPHPGGLLTIRLSVETISTQVGPNKVLAPLV

PTKNPGISRDKNTAGGTAGTHPKTSVTPSTPATQTTEDSLRKLVRTVYETLNATSPNL

TTSCWLCYDVKPPFYEAIGLNATYHASNGKNPSQCSWGNHKIGLSMQLVSGHVTCQGK

EPQAKQSLCASIDSSPSWKSDTKWLIPRTDGWSICSTTGFTPYLFTLVLNAANEFCVL

VTVQPRILYHPEESMYLHWDSDTSMRSKREPITAVTIATLFSLGIAGAGTGIASLATQ

QSGMTSLRAAIDEDIERLETSISHLEKSLTSLSEVVLQNRRGLDLLFLQQGGLCAALG

EECCFYADHTGVVKESMAKVREGLAKRKREREAQENWFEAWFNRSPWLTTLVSTLVGP

IILLVLILTLGPCILNKLINFVKDRVNTVQLMVLRQQYETVPTREDLYGWPVHEQDSS

L"

ORIGIN

1 cccttggctt taagtgtttg gggtccttgt taaaacccgc tgcgtcgggc agagactcgg

61 accccagctt gctggaaata aacctcgctg tgtgacttgc attattgtgt gggttctctg

121 tctgtctgag ggggacaatt ccggacctta acaggtgcgg caacgtccag gatgcccccc

181 cctcagggga acagacaact cccgataccg aggtttacat agggaaccgc ggaaggaggt

241 ttggccacct ccaatagggg aggactgaaa caggtcctgg accggagaga ctgaaacagg

301 tctctgcccg gtcccagttg agaattctca cgggaaggaa cgtgcgggtt acgaccctgg

361 aggctccgtt acttgaagtc gtgggagacg tccccgacga gaaggtggcc cagcgacgtc

421 cgcagtggac gccatttgga cgggccctgg gtaaggatct tgagcgtgtt gagactggtg

481 tgattgaaac ggcgcctgtg gatgtggtgt ggttgactgg ccggtgggtg ttgaagagtc

541 ccgtgcgaag ttgtgtattt gctggcactg ggtcttttgt cctttctttt gccttttctt

601 ggtttctctt tgtgacaatt atgggacagg ttcaggtaac acctaagacc ctgctcctga

661 accactttcc tgaaatccgc gccaaggctc gtaatcatgg tgtggaagtg aagaaaggta

721 agtttgatac attctgctct gcagaatggc ctacttttaa tgtgggctgg cccccccagg

781 gaacttttcc cctagacatt attaagaagg tccgagatat tattaatcgg cgccatccgg

841 accaataccc ctatattttg atatggcaag ccttagtaga gagtcctccc tcctggctta

901 agccctttat ccccgacaag ccagaaggtc ccccctccc ccttaaagtc ctgaccgttt

961 cgggaccctc ccgccagccg gcggtgccca cggccggccc gaaacccccg gagaagccga

1021 cccagggacc catccttcag gaggggtcag acaaataccc ctccctgata gacctagatc

1081 tggaagagac cccccctcct tacgcgccgg tggcgccgct ccagccacgg cgcgcgccca

1141 gcccagtggc gccgcttttg cctgaggctg ccgcccctcc cgagctgcct cactctaccg

1201 cctccccgat ggctccccct cccccgggtt cccctgcccc agctcaaggg ccggcgaggg

1261 gattgaagcc tcgtagacgc cgggaagaga ccccagagga ggaaccgtcc tcctccacct

1321 ccgctggggc gccgattctc cccgtgcgag cactaggagg aactggtcca gatggggagc

1381 gagcatacca gtactggccc ttttctagca gtgatctgta caactggaag gctcaaaacc

1441 ctcctttttc tgaggacccg aaaggtctaa ctaacctgtt cgagtctgtc atgcacacac

1501 acagtcccac ttgggatgat tgccagcagc ttcttaagac cttattcacc accgaggagc

1561 gcgagcgaat cctcactgag gccagaaaga atgtccccgg cgacaacggg cggccgacga

1621 ccttgccgaa cctgatcgac gagcgctttc ccctgaatag actggattgg gactttggga

1681 acgcagaagg tagggagcgt ctccgagtct accgccagac tcttatggca ggtctccgag

1741 cggcggcacg ccgccccacc aatttggcca aggtaaaagc tataatgcaa ggggaaaatg

1801 aaagcccggc cgtgttttta gaacgcctct atgatgctta tagacagtac accccgttgg

1861 accccctcgc agaggaaaac cagtcggctg taattatgtc ctttataaac caggctgccc

1921 cagatattag gaagaaattg tacaaacagg agggactggg agaaatgtcc attcgggatt

1981 taatgaaagt agcggagaga gtcttcaaca ctcgagagac tcccgaaaaa agggaggata

2041 gaattagaaa agaaaatcag gaattacagg aacgaatcag gaaggaagac agagagcatc

2101 agagcaggga aaacaggagg cagcagaggg agatggctaa gatcttgttg gcaggtgtgc

2161 aaagcacagt cagggtggga ccgagtccgg caggaccagc ccgaccgtgg agaccgcggc

2221 cccgactgga taggggacag tgtgcaaact gcaaggagta tggacattgg aagagggagt

2281 gccccaagcg ccagggccaa acagggcaag acgcacgggt cctgctggcg ggatggaga

2341 gtgactaggg gagacgggac tcggatcccc tccccgagtc ttgggtaact gcgtatgtgg

2401 aggggaagcc agtaggattc atggtagaca caggagccca gtactcagtt ttgaataagc

2461 ctacagagcc cttatctcag aaaaccagtt tggtacaagg ggcaactggg tccaaggctt

2521 atcggtggac tagtaagcgc caggtggact taggccgcca ccaagtgacc cactccttcc

2581 tagttatccc tgaatgccct gcccccttac tggggcgcga tctcctgact aagatcaggg

2641 ctcagatcca ttttgagccg gatggcatta agctattgga tggccaagga cagcccctcc

2701 acattttgac cctgtctctt gtggatgaac atcgcctgtt cgccctgcag gacaacccct

2761 acaaccctcc ctctacagaa tggccccgtg atatggatta ttggcttaaa acataccctc

2821 aggcgtgggc ggaaatagcg ggtgtgggcc gggcggcccg ccgagcacca gtagtggtgg

2881 aacttaaagc ctcggcccgg cctatccgga tccgccagta ccccatgtct gcagaggcgc

2941 ggaaagggat tgccccgcac attaaccgtt tactggaagc tggaatactg aaaccttgcc

3001 attctgcctg gaacacccca cttctccccg ttaagaaacc gggggggaaaa gattataggc

3061 cagtccagga cttgagggaa gtgaataaga gggttgaaga catccacccc acggtcccca

3121 acccttatac cttactaagt cacttgcccc cttcacatgt ctggtatact accttagacc

3181 taaaggatgc gttttttagc atagccctgg cacccagcag ccaacacatt tttgccttcg

3241 aatggaataa tggcaatacg ggaacccccg ggcagctgac ctggactaga ctaccgcaag

3301 gcttcaaaaa ctctccaact ctgtttaatg aagccctaaa tcaggatttg gactcgtttc

3361 gccagagcca taattcagtt acgctcctgc agtacgtaga tgacttgctt ctggcggccc

3421 cctccgaagc tgaatgccga caggccactg gagacctcct ccaggagctg gggcagttgg

3481 gctatcgggc cagtgcaaag aaggctcaaa tatgcaggca aacagtcacc tacctggggt

3541 ataaactaaa agaaggaacc agatggctga cagaggccat gaaagagact attcttagac

3601 ttccagtccc gacctcacca caagaggtcc gtgaattttt agggacgaca ggctactgcc

3661 ggctgtggat tttggggtat gctgaaatag caaaacctct gtatgaggca accaaggata

3721 aggtcccttg ggcctggggg tcagaccaac aaaaggccta cgatgaactc aaggtcgctc

3781 tcctaagagc ccggctctg gcattgccag accccctgaa gcccttcact ctctttgttg

3841 atgagaggag gggaatagcg aaaggggtgc taatgcagcg tctggggccc tggaaacgtc

3901 cggttgccta tttatccaag aagctagatc cagttgcagc aggatggccc ccgtgcttaa

3961 ggatcattgc ggcagtagcc ctaatggtga aggatgctga taaactcact tttgggcaac

4021 atctgaaggt agtgaccccc catgcgatcg agggggtcct gaaatatccc cctggtaggt

4081 ggatgactaa tgcccgacta acccattacc aaggactctt gctagatgca ccccggatca

4141 tcttcgctga acccaccgct ctgaatccag ccaccctgct gccgaccccg gatctgagag

4201 ctcccctgca tgattgccaa gagatcatgg cagaagtcac ccaggtgcgc cccgacctcc

4261 aggacaccgc actacccaac agtgagttgg tatggtacac tgatggaagc agcttcgtta

4321 tagatggtgt gcggagggca ggcgcagcgg tggtagacca agggggaaac atcatttgga

4381 atgcctcgct ttccccgggg acatcagcac agaaggccga actgatcgcg ctggcggagg

4441 cgctggaacg ggccaaaggg agacgagtga ctgtctacac cgatagccgc tacgcctttg

4501 gcactgtcca tgtgcatggc gctatctacc gggaaagagg ctttgttaca gcggaaggaa

4561 aggctctgcg caatcttcct gaggtacgaa gactgctgat ggctgtgcaa atgccccggg

4621 cagtcgcagt tgtccacatc cctgggcacc agtctgccca gaccccggaa gctgaaggaa

4681 accggcgagc ggatgaagcc gccaaggcag tggcagtagc ttcatcagct ttagcactca

4741 ccctgcccac acccgagctc cctcgcctgc ccccgcgacc tgactacact ccggaagacc

4801 tgcgatggat ccagaaccac cactgcccgg aatctgatca gcaggggtgg catcgggata

4861 cagaaagaag attgatactg ccggcacagc tagactgttt cttctctcca acctgcatca

4921 agccacccac ttaggaaaaa agaagttgct gacaattctc gagtccgccc gcctccggtt

4981 tccccgacaa gcggctcaga ttcaagagat tgtaaatcat ggcattgggt gccaggctat

5041 gagacccagt aggaaaggac cccaacatac aggtacgagg gtacggggaa gagcgccggg

5101 acggagttgg gaagtggatt ttactgaggt aaagcctggg aggtatgggt ataagtactt

5161 gctagtaatg gttgacacat tttcgggctg ggtggaagcc ttccccacga aacgggagac

5221 tgcccaagtg gttgctaagg cattactaga agaaattatt cccagatatg ggttcctga

5281 ggttttaggc tccgataacg gcccagcttt catcagtaac gtcctacagg gactagcccg

5341 ggcgataggg atcaattgga agttacattg tgaatataat ccccagagct cagggcaggt

5401 agagagaatg aatcggactc taagaggaga cctttgtcca aactagccat cgagactggc

5461 ggggactggg tgaccctctt accctatgcc atcttccggg tccggaactc accatatgta

5521 catggtttga cacctttcga aattctgtat ggggcaccac cccccattat tgttcgtact

5581 ctaccagatc atgaccccaa tgtggcccca agttatctgg ccagtttaaa ggccctacaa

5641 ggggtccaac atgagatatg gcccctagtg agttccctgt atgaaattaa ggacgccccg

5701 aacccggaac atggcatcgt tccaggggat tgggtatggg taaggagaca caggtcccgg

5761 acactggagg aaagatggaa aggtccttat gtggttattc tggttacccc cactgcctta

5821 aaggttgacg gcattgggcc ttgggtccat cactctcacg tgcgccgagc cagccagctg

5881 gagaagacgc aagctaagga gtggatcgta cggcgacacc ctgataaccc tctgaagctg

5941 cagctctccc gacctcgggg aagcgtcaag ccccatgcct cagctaacga tggaatggct

6001 gctagcccta actctgctca acatctggga gaagagccac gcggggatca acccacacca

6061 accccataag ctaacatgga ccctaacaga tggacagacc caaacaaccc ttaatagcac

6121 cacacatact gcccccatca atacgtggtg gccagatttg tttttcgacc tgcgtgacat

6181 tttcggcact aaacgtggac ggcagtatga ctactcagtc aggtccaagc gggctgtaat

6241 tgacacctct caaggacata gtgcacaagg gttttgggcc tgcccaggga acctaagaaa

6301 caattggaaa acctgtggcg gcccagaccg ctactattgg ggtagttgga gttgggtcac

6361 ctcctatgac gggccccgac agtgggacgt tgggaacagg gatctagtta aattctcctt

6421 tagggacccc cacaaccggg tgccccaggt atgtgtccag tttaaccaag acgtggcacg

6481 aagagagcgt ggttggttat caggattaac ttgggggttc caattagata taggccgttg

6541 ggcatggata ggcccccacc ccggcggtct cctaactatt cgactatcgg tggaaacgat

6601 cagcactcag gtgggtccaa ataaggtgct ggcccctctc gtccctacca agaacccagg

6661 tatatcaagg gataaaaaca ccgcaggagg gactgcggga acccacccaa agacctctgt

6721 aactccttcg acccctgcta cgcagacaac cgaagactca ttgcggaaac tggtgcgcac

6781 tgtatacgag acccttaatg ccaccagtcc taacctcaca acctcctgtt ggctgtgcta

6841 tgatgtaaag cccccgttct atgaggcaat aggacttaat gccacttacc acgcctctaa

6901 cgggaagaac ccttctcagt gttcatgggg gaatcataaa attggcttat ccatgcaact

6961 agtgagcggt catgttacct gtcaagggaa ggagccccag gctaaacaaa gtttatgtgc

7021 ctccatagac agctccccta gttggaaaag tgacactaag tggttaatcc ccagaactga

7081 tggatggtcg atatgttcaa cgactggctt caccccgtac ctatttacct tggtccttaa

7141 tgccgccaat gaattttgtg tcctagtaac agtgcagccc cgcatcctct atcaccctga

7201 ggagagtatg tatttgcatt gggatagtga cacaagtatg agaagtaaaa gagagcccat

7261 caccgcagta accattgcca ccctgttcag tttgggaata gccggagccg ggaccggcat

7321 agcttccctg gctactcaac aatcaggaat gacctcccta agggcggcca tagatgagga

7381 catagaaagg ttggaaacct cgattagtca tttagagaag tcgctcacct ctctatccga

7441 ggtagtactc cagaatagaa gaggacttga tttgttgttc ctccaacaag ggggactgtg

7501 tgccgcgctg ggggaggaat gttgttttta tgctgaccat actggtgtgg taaaagaatc

7561 catggcaaag gtgagagaag ggttagctaa gagaaaacgg gaaagggaag ctcaggagaa

7621 ctggtttgag gcttggttta acagatcccc ctggctcacc accttagtat ctaccttagt

7681 gggcccaatt atcttgcttg tgcttattct aaccttgggc ccttgcatat taaacaagct

7741 tattaatttt gtaaaagatc gtgttaatac cgtccagctc atggtcctaa gacaacagta

7801 tgagacagtg cccacccgtg aggacctcta cggctggccc gtacatgagc aagattcctc

7861 attatgaaca acacacaggg gggaaatgta agagactgtt agcatgagaa ctacatcttt

7921 aggtcagggt taacagcccc tagcctgaaa cagcatagtg ggaaatgtaa gagactgtta

7981 gcatgagaac tacatcttta ggtcaggttt accagcccct agcctgaaac aacataatta

8041 taaaattcca ggatgtgggc agttacagca tggagacgag aagtcctgca gcttatctta

8101 tactcctggt ttccttgttc tgtagtaaat cttatactct ttgaagctat gcaagtcctg

8161 tagtttatct tatactcttt gaagctatgc aagtcctgta gtttatctag tactctctga

8221 agctatggaa aattactgag tgccttgaaa atgctatata acccctgggc tttaagtgtt

8281 gggggtcctg gtaaaaaccc gctgcgtcgg gcagagactc ggaccccagc tggctggaaa

8341 taaacctcgc tgtgtgactt gca

//

LOCUS OR761825 8363 bp RNA linear VRL 13-NOV-2023

DEFINITION Rhinolophus ferrumequinum retrovirus isolate Y4b.

ACCESSION OR761825

VERSION OR761825 GI:2619391282

KEYWORDS .

SOURCE Rhinolophus ferrumequinum retrovirus (RfRV)

ORGANISM Rhinolophus ferrumequinum retrovirus

Viruses; Riboviria; Pararnavirae; Artverviricota; Revtraviricetes;

Ortervirales; Retroviridae; Orthoretrovirinae; Gammaretrovirus; Bat
gammaretrovirus.

REFERENCE 1 (bases 1 to 8363)

AUTHORS Chung,H., Nguyen,V., Hwang,S., Chung,C. and Lee,J.

TITLE Identification of Rhinolophus ferrumequinum retrovirus of bats in
Korea

JOURNAL Unpublished

REFERENCE 2 (bases 1 to 8363)

AUTHORS Chung,H., Nguyen,V., Hwang,S., Chung,C. and Lee,J.

TITLE Direct Submission

JOURNAL Submitted (01-NOV-2023) Department of Microbiology and Immunology,
Institute for Immunology and Immunological Diseases, Yonsei
University College of Medicine, 50-1, Yonsei-ro, Seodaemun-gu,
Seoul Ks031, Korea

COMMENT ##Assembly-Data-START##
Assembly Method :: BioEdit v. v. 7.71
Sequencing Technology :: Illumina; Sanger dideoxy sequencing
##Assembly-Data-END##

FEATURES Location/Qualifiers

source 1..8363
/organism="Rhinolophus ferrumequinum retrovirus"
/mol_type="genomic RNA"
/isolate="Y4b"
/host="Rhinolophus ferrumequinum; bat"
/db_xref="taxon:1147756"
/country="South Korea"

/collection_date="2022-08-17"

CDS 621..2348

/codon_start=1

/product="gag protein"

/protein_id="WPA94625"

/db_xref="GI:-1675576013"

/translation="MGQVQVTPKTLLLNHFPEIRAKARNHGVEVKKGKFDTFCSAEWP

TFNVGWPPQGTFPLDIIKKVRDIINRRHPDQYPYILIWQALVESPPSWLKPFIPDKPE

GPPLPLKVLTVSGPSRQPAVPTAGPKPPEKPTQGPILQEGSDKYPSLIDLDLEETPPP

YAPVAPLQPRRAPSPVAPLLPEAAAPPELPHSTASPMAPPPPGSPAPAQGPARGLKPR

RRREETPEEEPASSTSAGAPILPVRALGGTGPDGERAYQYWPFSSSDLYNWKAQNPPF

SEDPKGLTNLFESVMHTHSPTWDDCQQLLKTLFTTEERERILTEARKKVPGDNGRPTT

LPNLIDERFPLNRLDWDFGNAEGRERLRVYRQTLMAGLRAAARRPTNLAKVKAIMQGE

NESPAVFLERLYDAYRQYTPLDPLAEENQSAVIMSFINQAAPDIRKKLYKQEGLGEMS

IRDLMKVAERVFNTRETPEKREDRIRKENQELQERIRKEDREHQSRENRRQQREMAKI

LLAGVQSTVRVGPSPAGPARPWRPRPRLDRGQCANCKEYGHWKRECPKRQGQTGQDAR

VLLAGMESD"

CDS <2349..4934

/codon_start=1

/product="pol protein"

/protein_id="WPA94626"

/db_xref="GI:-1675576012"

/translation="GRRDSDPLPESWVTAYVEGKPVGFMVDTGAQYSVLNKPTEPLSQ

KTSLVQGATGSKAYRWTSKRQVDLGRHQVTHSFLVIPECPAPLLGRDLLTKIRAQIHF

EPDGIKLLDGQGQPLHILTLSLVDEHRLFALQDNPYNPPSTEWPRDMDYWLKTYPQAW

AEIAGVGRAARRAPVVVELKASARPIRIRQYPMSAEARKGNAPHINRLLEAGILKPCH

SAWNTPLLPVKKPGGKDYRPVQDLREVNKRVEDIHPTVPNPYTLLSHLPPSHVWYTTL

DLKDAFFSIALAPSSQHIFAFEWNNGNTGTPGQLTWTRLPQGFKNSPTLFNEALNQDL

DSFRQSHNSVTLLQYVDDLLLAAPSEAECRQATGDLRQELGQLGYRASAKKAQICRQT

VTYLGYKLKEGTRWLTEAMKETILRLPVPTSPQEVREFLGTTGYCRLWILGYAEIAKP

LYEATKDKVPWAWGSDQQKAYDELKVALLRAPALALPDPLKPFTLFVDERRGIAKGVL

MQRLGPWKRPVAYLSKKLDPVAAGWPPCLRIIAAVALMVKDADKLTFGQHLKVVTPHA

IEGVLKYPPGRWMTNARLTHYQGLLLDAPRIIFAEPTALNPATLLPTPDLRAPLHDCQ

EIMAEVTQVRPDLQDTALPNSELVWYTDGSSFVIDGVRRAGAAVVDQGGNIIWTASLS

PGTSAQKAELIALAEALERAKGRRVTVYTDSRYAFGTVHVHGAIYRERGFVTAEGKAL

RNLPEVRRLLMAVQMPRAVAVVHIPGHQSAQTPEAEGNRRADEAAKAVAVASSALALT

LPTPELPRLPPRPDYTPEDLRWIQNHHCPESDQQGWHRDTERRLILPAQLDCFFSPTC

IKPPT"

CDS 5990..7867

/codon_start=1

/product="envelope glycoprotein"

/protein_id="WPA94627"

/db_xref="GI:-1675576011"

/translation="MEWLLALTLLNIWEKSHAGINPHQPHKLTWTLTDGQTQTTLNST

TNTAPINTWWPDLFFDLRDIFGTKRGRQYDYSVRSKRAVIDTSQGHSAQGFWACPGNL

RNNWKTCGGPDRYYWGSWSWVTSYDGPRQWDVGNRDLVKFSFRDPHNRVPQVCVQFNQ

DVARRERGWLSGLTWGFQLDIGRWAWIGPHPGGLLTIRLSVETISTQVGPNKVLAPLV

PTKNPGISRDKNTAGGTAGTHPKTSVTPSTPATQTTEDSLRKLVRTVYETLNATSPNL

TTSCWLCYDVKPPFYEAIGLNATYHASKGKNPSQCSWGNHKIGLSMQLVSGHVTCQGK

EPQAKQSLCASIDSSPSWKSDTKWLIPRTDGWSICSTTGFTPYLFTLVLNAANEFCVL

VTVQPRILYHPEESMYLHWDSATSMRSKREPITAVTIATLFSLGIAGAGTGIASLATQ

QSGMTSLRAAIDEDIERLETSISHLEKSLTSLSEVVLQNRRGLDLLFLQQGGLCAALG

EECCFYADHTGVVKESMAKVREGLAKRKREREAQENWFEAWFNRSPWLTTLVSTLVGP

IILLVLILTLGPCILNKLINFVKDRVNTVQLMVLRQQYETVPTREDLYGWPVHEQDSS

L"

ORIGIN

1 cccttggctt taagtgtttg gggtccttgt taaaacccgc tgcgtcgggc agagactcgg

61 accccagctt gctggaaata aacctcgctg tgtgacttgc attattgtgt gggttctctg

121 tctgtctgag ggggacaatt ccggacctta acaggtgcgg caacgtccag gatgcccccc

181 cctcagggga acagacaact cccgataccg aggtttacat agggaaccgc ggaaggaggt

241 ttggccacct ccaatagggg aggactgaaa caggtcctgg accggagaga ctgaaacagg

301 tctctgcccg gtcccagttg agaattctca cgggaaggaa cgtgcgggtt acgaccctgg

361 aggctccgtt acttgaagtc gtgggagacg tccccgacga gaaggtggcc cagcgacgtc

421 cgcagtggac gccatttgga cgggccctgg gtaaggatct tgagcgtgtt gagactggtg

481 tgattgaaac ggcgcctgtg gatgtggtgt ggttgactgg ccggtgggtg ttgaagagtc

541 ccgtgcgaag ttgtgtattt gctggcactg ggtcttttgt cctttctttt gccttttctt

601 ggtttctctt tgtgacaatt atgggacagg ttcaggtaac acctaagacc ctgctcctga

661 accactttcc tgaaatccgc gccaaggctc gtaatcatgg tgtggaagtg aagaaaggta

721 agtttgatac attctgctct gcagaatggc ctacttttaa tgtgggctgg cccccccagg

781 gaacttttcc cctagacatt attaagaagg tccgagatat tattaatcgg cgccatccgg

841 accaataccc ctatattttg atatggcaag ccttagtaga gagtcctccc tcctggctta

901 agccctttat ccccgacaag ccagaaggtc ccccctccc ccttaaagtc ctgaccgttt

961 cgggaccctc ccgccagccg gcggtgccca cggccggccc gaaacccccg gagaagccga

1021 cccagggacc catccttcag gaggggtcag acaaataccc ctccctgata gacctagatc

1081 tggaagagac cccccctcct tacgcgccgg tggcgccgct ccagccacgg cgcgcgccca

1141 gcccagtggc gccgcttttg cctgaggctg ccgcccctcc cgagctgcct cactctaccg

1201 cctccccgat ggctccccct cccccgggtt cccctgcccc agctcaaggg ccggcgaggg

1261 gattgaagcc tcgtagacgc cgggaagaga ccccagagga ggaaccggcc tcctccacct

1321 ccgctggggc gccgattctc cccgtgcgag cactaggagg aactggtcca gatggggagc

1381 gagcatacca gtactggccc ttttctagca gtgatctgta caactggaag gctcaaaacc

1441 ctccttttc tgaggacccg aaaggtctaa ctaacctgtt cgagtctgtc atgcacacac

1501 acagtcccac ttgggatgat tgccagcagc ttcttaagac cttattcacc accgaggagc

1561 gcgagcgaat cctcactgag gccagaaaga aagtccccgg cgacaacggg cggccgacga

1621 ccttgccgaa cctgatcgac gagcgctttc ccctgaatag actggattgg gactttggga

1681 acgcagaagg tagggagcgt ctccgagtct accgccagac tcttatggca ggtctccgag

1741 cggcggcacg ccgccccacc aatttggcca aggtaaaagc tataatgcaa ggggaaaatg

1801 aaagcccggc cgtgttttta gaacgcctct atgatgctta tagacagtac accccgttgg

1861 accccctcgc agaggaaaac cagtcggctg taattatgtc ctttataaac caggctgccc

1921 cagatattag gaagaaattg tacaaacagg agggactggg agaaatgtcc attcgggatt

1981 taatgaaagt agcggagaga gtcttcaaca ctcgagagac tcccgaaaaa agggaggata

2041 gaattagaaa agaaaatcag gaattacagg aacgaatcag gaaggaagac agagagcatc

2101 agagcaggga aaacaggagg cagcagaggg agatggctaa gatcttgttg gcaggtgtgc

2161 aaagcacagt cagggtggga ccgagtccgg caggaccagc ccgaccgtgg agaccgcggc

2221 cccgactgga taggggacag tgtgcaaact gcaaggagta tggacattgg aagagggagt

2281 gccccaagcg ccagggccaa acagggcaag acgcacgggt cctgctggcg gggatggaga

2341 gtgactaggg gagacgggac tcggatcccc tccccgagtc ttgggtaact gcgtatgtgg

2401 aggggaagcc agtaggattc atggtagaca caggagccca gtactcagtt ttgaataagc

2461 ctacagagcc cttatctcag aaaaccagtt tggtacaagg ggcaactggg tccaaggctt

2521 atcggtggac tagtaagcgc caggtggact taggccgcca ccaagtgacc cactccttcc

2581 tagttatccc tgaatgccct gcccccttac tggggcgcga tctcctgact aagatcaggg

2641 ctcagatcca ttttgagccg gatggcatta agctattgga tggccaagga cagcccctcc

2701 acattttgac cctgtctctt gtggatgaac atcgcctgtt cgccctgcag gacaacccct

2761 acaaccctcc ctctacagaa tggccccgtg atatggatta ttggcttaaa acataccctc

2821 aggcgtgggc ggaaatagcg ggtgtgggcc gggcggcccg ccgagcacca gtagtggtgg

2881 aacttaaagc ctcggcccgg cctatccgga tccgccagta ccccatgtct gcagaggcgc

2941 ggaaagggaa tgccccgcac attaaccgtt tactggaagc tggaatactg aaaccttgcc

3001 attctgcctg gaacacccca cttctccccg ttaagaaacc gggggaaaa gattataggc

3061 cagtccagga cttgagggaa gtgaataaga gggttgaaga catccacccc acggtcccca

3121 acccttatac cttactaagt cacttgcccc cttcacatgt ctggtatact accttagacc

3181 taaaggatgc gttttttagc atagccctgg cacccagcag ccaacacatt tttgccttcg

3241 aatggaataa tggcaatacg ggaacccccg ggcagctgac ctggactaga ctaccgcaag

3301 gcttcaaaaa ctctccaact ctgtttaatg aagccctaaa tcaggatttg gactcgtttc

3361 gccagagcca taattcagtt acgctcctgc agtacgtaga tgacttgctt ctggcggccc

3421 cctccgaagc tgaatgccga caggccactg gagacctccg ccaggagctg ggcagttgg

3481 gctatcgggc cagtgcaaag aaggctcaaa tatgcaggca aacagtcacc tacctggggt

3541 ataaactaaa agaaggaacc agatggctga cagaggccat gaaagagact attcttagac

3601 ttccagtccc gacctcacca caagaggtcc gtgaattttt agggacgaca ggctactgcc

3661 ggctgtggat tttggggtat gctgaaatag caaaacctct gtatgaggca accaaggata

3721 aggtcccttg ggcctggggg tcagaccaac aaaaggccta cgatgaactc aaggtcgctc

3781 tcctaagagc cccggctctg gcattgccag accccctgaa gcccttcact ctctttgttg

3841 atgagaggag gggaatagcg aaaggggtgc taatgcagcg tctggggccc tggaaacgtc

3901 cggttgccta tttatccaag aagctagatc cagttgcagc aggatggccc ccgtgcttaa

3961 ggatcattgc ggcagtagcc ctaatggtga aggatgctga taaactcact tttgggcaac

4021 atctgaaggt agtgaccccc catgcgatcg agggggtcct gaaatatccc cctggtaggt

4081 ggatgactaa tgcccgacta acccattacc aaggactctt gctagatgca ccccggatca

4141 tcttcgctga acccaccgct ctgaatccag ccaccctgct gccgaccccg gatctgagag

4201 ctcccctgca tgattgccaa gagatcatgg cagaagtcac ccaggtgcgc cccgacctcc

4261 aggacaccgc actacccaac agtgagttgg tatggtacac tgatggaagc agcttcgtta

4321 tagatggtgt gcggagggca ggcgcagcgg tggtagacca agggggaaac atcatttgga

4381 ctgcctcgct ttccccgggg acatcagcac agaaggccga actgatcgcg ctggcggagg

4441 cgctggaacg ggccaaaggg agacgagtga ctgtctacac cgatagccgc tacgcctttg

4501 gcactgtcca tgtgcatggc gctatctacc gggaaagagg ctttgttaca gcggaaggaa

4561 aggctctgcg caatcttcct gaggtacgaa gactgctgat ggctgtgcaa atgccccggg

4621 cagtcgcagt tgtccacatc cctgggcacc agtctgccca gaccccggaa gctgaaggaa

4681 accggcgagc ggatgaagcc gccaaggcag tggcagtagc ttcatcagct ttagcactca

4741 ccctgcccac acccgagctc cctcgcctgc ccccgcgacc tgactacact ccggaagacc

4801 tgcgatggat ccagaaccac cactgcccgg aatctgatca gcaggggtgg catcgggata

4861 cagaaagaag attgatactg ccggcacagc tagactgttt cttctctcca acctgcatca

4921 agccacccac ttaggaaaaa agaagttgct gacaattctc gagtccgccc gcctccggtt

4981 tccccgacaa gcggctcaga ttcaagagat tgtaaatcat ggcattgggt gccaggctat

5041 gagacccagt aggaaaggac cccaacatac aggtacgagg gtacggggaa gagcgccggg

5101 acggagttgg gaagtggatt ttactgaggt aaagcctggg aggtatgggt ataagtactt

5161 gctagtaatg gttgacacat tttcgggctg ggtggaagcc ttccccacga aacgggagac

5221 tgcccaagtg gttgctaagg cattactaga agaaattatt cccagatatg gggttcctga

5281 ggttttaggc tccgataacg gcccagcttt catcagtaac gtcctacagg gactagcccg

5341 ggcgataggg atcaattgga agttacattg tgaatataat ccccagagct cagggcaggt

5401 agagagaatg aatcggactc taagaggaga cctttgtcca aactagccat cgagactggc

5461 ggggactggg tgaccctctt accctatgcc atcttccggg tccggaactc accatatgta

5521 catggtttga cacctttcga aattctgtat ggggcaccac cccccattat tgttcgtact

5581 ctaccagatc atgaccccaa tgtggcccca agttatctgg ccagtttaaa ggccctacaa

5641 ggggtccaac atgagatatg gcccctagtg agttccctgt atgaaattaa ggacgccccg

5701 aacccggaac atggcatcgt tccaggggat tgggtatggg taaggagaca caggtcccgg

5761 acactggagg aaagatggaa aggtccttat gtggttattc tggttacccc cactgcctta

5821 aaggttgacg gcattgggcc ttgggtccat cactctcacg tgcgccgagc cagccagctg

5881 gagaagacgc aagctaagga gtggatcgta cggcgacacc ctgataaccc tctgaagctg

5941 cagctctccc gacctcgggg aagcgtcaag ccccatgcct cagctaacga tggaatggct

6001 gctagcccta actctgctca acatctggga gaagagccac gcggggatca acccacacca

6061 accccataag ctaacatgga ccctaacaga tggacagacc caaacaaccc ttaatagcac

6121 cacaaatact gcccccatca atacgtggtg gccagatttg tttttcgacc tgcgtgacat

6181 tttcggcact aaacgtggac ggcagtatga ctactcagtc aggtccaagc gggctgtaat

6241 tgacacctct caaggacata gtgcacaagg gttttgggcc tgcccaggga acctaagaaa

6301 caattggaaa acctgtggcg gcccagaccg ctactattgg ggtagttgga gttgggtcac

6361 ctcctatgac gggccccgac agtgggacgt tgggaacagg gatctagtta aattctcctt

6421 tagggacccc cacaaccggg tgccccaggt atgtgtccag tttaaccaag acgtggcacg

6481 aagagagcgt ggttggttat caggattaac ttgggggttc caattagata taggccgttg

6541 ggcatggata ggcccccacc ccggcggtct cctaactatt cgactatcgg tggaaacgat

6601 cagcactcag gtgggtccaa ataaggtgct ggcccctctc gtccctacca agaacccagg

6661 tatatcaagg gataaaaaca ccgcaggagg gactgcggga acccacccaa agacctctgt

6721 aactccttcg acccctgcta cgcagacaac cgaagactca ttgcggaaac tggtgcgcac

6781 tgtatacgag acccttaatg ccaccagtcc taacctcaca acctcctgtt ggctgtgcta

6841 tgatgtaaag cccccgttct atgaggcaat aggacttaat gccacttacc acgcctctaa

6901 agggaagaac ccttctcagt gttcatgggg gaatcataaa attggcttat ccatgcaact

6961 agtgagcggt catgttacct gtcaagggaa ggagccccag gctaaacaaa gtttatgtgc

7021 ctccatagac agctccccta gttggaaaag tgacactaag tggttaatcc ccagaactga

7081 tggatggtcg atatgttcaa cgactggctt caccccgtac ctatttacct tggtccttaa

7141 tgccgccaat gaattttgtg tcctagtaac agtgcagccc cgcatcctct atcaccctga

7201 ggagagtatg tatttgcatt gggatagtgc cacaagtatg agaagtaaaa gagagcccat

7261 caccgcagta accattgcca ccctgttcag tttgggaata gccggagccg ggaccggcat

7321 agcttccctg gctactcaac aatcaggaat gacctcccta agggcggcca tagatgagga

7381 catagaaagg ttggaaacct cgattagtca tttagagaag tcgctcacct ctctatccga

7441 ggtagtactc cagaatagaa gaggacttga tttgttgttc ctccaacaag ggggactgtg

7501 tgccgcgctg ggggaggaat gttgttttta tgctgaccat actggtgtgg taaaagaatc

7561 catggcaaag gtgagagaag ggttagctaa gagaaaacgg gaaagggaag ctcaggagaa

7621 ctggtttgag gcttggttta acagatcccc ctggctcacc accttagtat ctaccttagt

7681 gggcccaatt atcttgcttg tgcttattct aaccttgggc ccttgcatat taaacaagct

7741 tattaatttt gtaaaagatc gtgttaatac cgtccagctc atggtcctaa gacaacagta

7801 tgagacagtg cccacccgtg aggacctcta cggctggccc gtacatgagc aagattcctc

7861 attatgaaca acacacaggg gggaaatgta agagactgtt agcatgagaa ctacatcttt

7921 aggtcagggt taacagcccc tagcctgaaa cagcatagtg ggaaatgtaa gagactgtta

7981 gcatgagaac tacatcttta ggtcaggttt accagcccct agcctgaaac aacataatta

8041 taaaattcca ggatgtgggc agttacagca tggagacgag aagtcctgca gcttatctta

8101 tactcctggt ttccttgttc tgtagtaaat cttatactct ttgaagctat gcaagtcctg

8161 tagtttatct tatactcttt gaagctatgc aagtcctgta gtttatctag tactctctga

8221 agctatggaa aattactgag tgccttgaaa atgctatata acccctgggc tttaagtgtt

8281 gggggtcctg gtaaaaaccc gctgcgtcgg gcagagactc ggaccccagc tggctggaaa

8341 taaacctcgc tgtgtgactt gca

//

LOCUS OR761826 8363 bp RNA linear VRL 13-NOV-2023

DEFINITION Rhinolophus ferrumequinum retrovirus isolate Y6a.

ACCESSION OR761826

VERSION OR761826 GI:2619391286

KEYWORDS .

SOURCE Rhinolophus ferrumequinum retrovirus (RfRV)

ORGANISM Rhinolophus ferrumequinum retrovirus

Viruses; Riboviria; Pararnavirae; Artverviricota; Revtraviricetes;

Ortervirales; Retroviridae; Orthoretrovirinae; Gammaretrovirus; Bat

gammaretrovirus.

REFERENCE 1 (bases 1 to 8363)

AUTHORS Chung,H., Nguyen,V., Hwang,S., Chung,C. and Lee,J.

TITLE Identification of Rhinolophus ferrumequinum retrovirus of bats in

Korea

JOURNAL Unpublished

REFERENCE 2 (bases 1 to 8363)

AUTHORS Chung,H., Nguyen,V., Hwang,S., Chung,C. and Lee,J.

TITLE Direct Submission

JOURNAL Submitted (01-NOV-2023) Department of Microbiology and Immunology,

Institute for Immunology and Immunological Diseases, Yonsei

University College of Medicine, 50-1, Yonsei-ro, Seodaemun-gu,

Seoul Ks031, Korea

COMMENT ##Assembly-Data-START##

Assembly Method :: BioEdit v. v. 7.71

Sequencing Technology :: Illumina; Sanger dideoxy sequencing

##Assembly-Data-END##

FEATURES Location/Qualifiers

source 1..8363

/organism="Rhinolophus ferrumequinum retrovirus"

/mol_type="genomic RNA"

/isolate="Y6a"

/host="Rhinolophus ferrumequinum; bat"

/db_xref="taxon:1147756"

/country="South Korea"

/collection_date="2023-05-20"

CDS 621..2348

/codon_start=1

/product="gag protein"

/protein_id="WPA94628"

/db_xref="GI:-1675576009"

/translation="MGQVQVTPKTLLLNHFPEIRAKARNQGVEVKKGKFDPFCSAEWP

TFNVGWPPQGTFPLDIIKKVRDIINRRHPDQYPYILIWQALVESPPSWLKPFIPDKPE

GPPLPLKVLTVSGPSRQPAVPTAGPKPPEKPTQGPILQEGSDKYPSLIDLDLEETPPP

YAPVAPLQPRRAPSPVAPLLPEAAAPPELPHSTASPMAPPPPGSPAPAQGPARGLKPR

RRREETPEEEPSSSTSAGAPILPVRALGGTGPDGERAYQYWPFSSSDLYNWKAQNPPF

SEDPKGLTNLFESVMHTHSPTWDDCQQLLKTLFTTAERERIPTEARKNVPGDNGRPTT

LPNLIDERFPLNRLDWDFGNAEGRERLRVYRQTLMAGLRAAARRPTNLAKVKAIMQGE

NESPAVFLERLYDAYRQYTPLDPLAEENQSAVIMSFINQAAPDIRKKLYKQEGLGEMS

LRDLMKVAERVFNTRETPEKREDRIRKENQELQERIRKEDREHQSRENRRQQREMAKI

LLAGVQSTVRVGPSPAGPARPWRPRPRLDRGQCANCKEYGHWKRECPKRQGQTGQDAR

VLLAGMESD"

CDS <2349..4934

/codon_start=1

/product="pol protein"

/protein_id="WPA94629"

/db_xref="GI:-1675576008"

/translation="GRRDSDPLPESWVTAYVEGKPVGFMVDTGAQYSVLNKPTEPLSQ

KTSLVQGATGSKAYRWTSKRQVDLGRHQVTHSFLVIPECPAPLLGRDLLTKNRAQIHF

EPDGIKLLDGQEQPLHILTLSLVDEHRLFALQDNPYNPPSTEWPRDMDYWLKTYPQAW

AEIAGVGRAARRAPVVVELKASARPIRIRQYPMSAEARKGIAPHINRLLEAGILKPCH

SAWNTPLLPVKKPGGKDYRPVQDLREVNKRVEDIHPTVPNPYTLLSHLPPSHVWYTTL

DLKDAFFSIALAPSSQHIFAFEWNNGNTGTPGQLTWTRLPQGFKNSPTLFNEALNQDL

DSFRQSHNSVTLLQYVDDLLLAAPSEAECRQATGDLLQELWQLGYRASAKKAQICRQT

VTYLGYKLKEGTRWLTEAMKETILRLPVPTSPQEVREFLGTTGYCRLWILGYAEIAKP

LYEATKDKVPWAWGSDQQKAYDELKVALLRAPALALPDPLKPFTLFVDERRGIAKGVL

MQRLGPWKRPVAYLSKKLDPVAAGWPPCLRIIAAVALMVKDADKLTFGQHLKVVTPHA

IEGVLKYPPGRWMTNARLTHYQGLLLDAPRIIFAEPTALNPATLLPTPDLRAPLHDCQ

EIMAEVTQVRPDLQDTALPNSELVWYTDGSSFVIDGVRRAGAAVVDQGGNIIWNASLS

PGTSAQKAELIALAEALERAKGRRVTVYTDSRYAFGTVHVHGAIYRERGFVTAEGKAL

RNLPEVRRLLMAVQMPRAVAVVHIPGHQSAQTPEAEGNRRADEAAKAVAVASSALALT

LPTPELPRLPPRPDYTPEDLRWIQNHHCPESDQQGWHRDTERRLILPAQLDCFFSPTC

IKPPT"

CDS 5990..7867

/codon_start=1

/product="envelope glycoprotein"

/protein_id="WPA94630"

/db_xref="GI:-1675576007"

/translation="MEWLLALTLLNIWEKTHAGINPHQPQKLTWTLTDGQTQTTLNST

THTAPINTWWPDLFFDLRDIFGTKRGRQYDYSVRSKRAVIDTSQGHNAQGFWACPGDL

RNNWKTCGGPDRYYWGSWSWVTSYDGPRQWDVGNRDLVKFSFRDPHNRVPQVCVQFNQ

DVARRERGWLSGLTWGFQLDIGRLAWIGPHPGGLLTIRLSVETISTQVGPNKVLAPLV

PTKNPGISRDKNTAGGTAGTHPKTSVTPSTPATQTTEDSLRKLVRTVYETLNATSPNL

TTSCWLCYDVKPPFYEAIGLNATYHASNGKNPSQFSWGNHKIGLSMQLVSGHVTCQGK

EPQAKQSLCASIDSSPSWKSDTKWLIPRTDGWSICSTTGFTPYLFTLVLNAANEFCVL

VTVQPRILYPPEESMYLHWDSDTSMRSKREPITAVTIATLFSLGIAGAGTGIASLATQ

QSGMTSLRAAIDEDIERLETSISHLEKSLTSLSEVVLQNRRGLDLLFLQQGGLCAALG

EECCFYADHTGVVKESMAKVREGLAKRKREREAQENWFEAWFNRSPWLTTLVSTLVGP

IILLVLILTLGPCILNKLINFVKDRVNTVQLMVLRQQYETVPTREDLYGWPVHEQDSS

L"

ORIGIN

1 cccttggctt taagtgtttg gggtccttgt taaaacccgc tgcgtcgggc agagactcgg

61 accccagctt gctggaaata aacctcgctg tgtgacttgc attattgtgt gggttctctg

121 tctgtctgag ggggacaatt ccggacctta acaggtgcgg caacgtccag gatgcccccc

181 cctcagggga acagacaact cccgataccg aggtttacat agggaaccgc ggaaggaggt

241 ttggccacct ccaatagggg aggactgaaa caggtcctgg accggagaga ctgaaacagg

301 tctctgcccg gtcccagttg agaattctca cgggaaggaa cgtgcgggtt acgaccctgg

361 aggctccgtt acttgaagtc gtgggagacg tccccgacga gaaggtggcc cagcgacgtc

421 cgcagtggac gccatttgga cgggccctgg gtaaggatct tgagcgtgtt gagactggtg

481 tgattgaaac ggcgcctgtg gatgtggtgt ggttgactgg ccggtgggtg ttgaagagtc

541 ccgtgcgaag ttgtgtattt gctggcactg ggtcttttgt cctttctttt gccttttctt

601 ggtttctctt tgtgacaatt atgggacagg ttcaggtaac acctaagacc ctgctcctga

661 accactttcc tgaaatccgc gccaaggctc gtaatcaagg tgtggaagtg aagaaaggta

721 agtttgatcc attctgctct gcagaatggc ctacttttaa tgtgggctgg cccccccagg

781 gaacttttcc cctagacatt attaagaagg tccgagatat tattaatcgg cgccatccgg

841 accaataccc ctatattttg atatggcaag ccttagtaga gagtcctccc tcctggctta

901 agccctttat ccccgacaag ccagaaggtc cccccctccc ccttaaagtc ctgaccgttt

961 cgggaccctc ccgccagccg gcggtgccca cggccggccc gaaacccccg gagaagccga

1021 cccagggacc catccttcag gaggggtcag acaaataccc ctccctgata gacctagatc

1081 tggaagagac ccccccctcct tacgcgccgg tggcgccgct ccagccacgg cgcgcgccca

1141 gcccagtggc gccgcttttg cctgaggctg ccgcccctcc cgagctgcct cactctaccg

1201 cctccccgat ggctccccct cccccgggtt cccctgcccc agctcaaggg ccggcgaggg

1261 gattgaagcc tcgtagacgc cgggaagaga ccccagagga ggaaccgtcc tcctccacct

1321 ccgctggggc gccgattctc cccgtgcgag cactaggagg aactggtcca gatggggagc

1381 gagcatacca gtactggccc ttttctagca gtgatctgta caactggaag gctcaaaacc

1441 ctcctttttc tgaggacccg aaaggtctaa ctaacctgtt cgagtctgtc atgcacacac

1501 acagtcccac ttgggatgat tgccagcagc ttcttaagac cttattcacc accgcggagc

1561 gcgagcgaat ccccactgag gccagaaaga atgtccccgg cgacaacggg cggccgacga

1621 ccttgccgaa cctgatcgac gagcgctttc ccctgaatag actggattgg gactttggga

1681 acgcagaagg tagggagcgt ctccgagtct accgccagac tcttatggca ggtctccgag

1741 cggcggcacg ccgccccacc aatttggcca aggtaaaagc tataatgcaa ggggaaaatg

1801 aaagcccggc cgtgttttta gaacgcctct atgatgctta tagacagtac accccgttgg

1861 accccctcgc agaggaaaac cagtcggctg taattatgtc ctttataaac caggctgccc

1921 cagatattag gaagaaattg tacaaacagg agggactggg agaaatgtcc cttcgggatt

1981 taatgaaagt agcggagaga gtcttcaaca ctcgagagac tcccgaaaaa agggaggata

2041 gaattagaaa agaaaatcag gaattacagg aacgaatcag gaaggaagac agagagcatc

2101 agagcaggga aaacaggagg cagcagaggg agatggctaa gatcttgttg gcaggtgtgc

2161 aaagcacagt cagggtggga ccgagtccgg caggaccagc ccgaccgtgg agaccgcggc

2221 cccgactgga taggggacag tgtgcaaact gcaaggagta tggacattgg aagagggagt

2281 gccccaagcg ccagggccaa acagggcaag acgcacgggt cctgctggcg ggatggaga

2341 gtgactaggg gagacgggac tcggatcccc tccccgagtc ttgggtaact gcgtatgtgg

2401 aggggaagcc agtaggattc atggtagaca caggagccca gtactcagtt ttgaataagc

2461 ctacagagcc cttatctcag aaaaccagtt tggtacaagg ggcaactggg tccaaggctt

2521 atcggtggac tagtaagcgc caggtggact taggccgcca ccaagtgacc cactccttcc

2581 tagttatccc tgaatgccct gcccccttac tggggcgcga tctcctgact aagaacaggg

2641 ctcagatcca ttttgagccg gatggcatta agctattgga tggccaagaa cagcccctcc

2701 acattttgac cctgtctctt gtggatgaac atcgcctgtt cgccctgcag gacaacccct

2761 acaaccctcc ctctacagaa tggccccgtg atatggatta ttggcttaaa acataccctc

2821 aggcgtgggc ggaaatagcg ggtgtgggcc gggcggcccg ccgagcacca gtagtggtgg

2881 aacttaaagc ctcggcccgg cctatccgga tccgccagta ccccatgtct gcagaggcgc

2941 ggaaagggat tgccccgcac attaaccgtt tactggaagc tggaatactg aaaccttgcc

3001 attctgcctg gaacacccca cttctccccg ttaagaaacc ggggggaaaa gattataggc

3061 cagtccagga cttgagggaa gtgaataaga gggttgaaga catccacccc acggtcccca

3121 acccttatac cttactaagt cacttgcccc cttcacatgt ctggtatact accttagacc

3181 taaaggatgc gttttttagc atagccctgg cacccagcag ccaacacatt tttgccttcg

3241 aatggaataa tggcaatacg ggaacccccg ggcagctgac ctggactaga ctaccgcaag

3301 gcttcaaaaa ctctccaact ctgtttaatg aagccctaaa tcaggatttg gactcgtttc

3361 gccagagcca taattcagtt acgctcctgc agtacgtaga tgacttgctt ctggcggccc

3421 cctccgaagc tgaatgccga caggccactg gagacctcct ccaggagctg tggcagttgg

3481 gctatcgggc cagtgcaaag aaggctcaaa tatgcaggca aacagtcacc tacctggggt

3541 ataaactaaa agaaggaacc agatggctga cagaggccat gaaagagact attcttagac

3601 ttccagtccc gacctcacca caagaggtcc gtgaattttt agggacgaca ggctactgcc

3661 ggctgtggat tttggggtat gctgaaatag caaaacctct gtatgaggca accaaggata

3721 aggtcccttg ggcctggggg tcagaccaac aaaaggccta cgatgaactc aaggtcgctc

3781 tcctaagagc ccggctctg gcattgccag accccctgaa gcccttcact ctctttgttg

3841 atgagaggag gggaatagcg aaaggggtgc taatgcagcg tctggggccc tggaaacgtc

3901 cggttgccta tttatccaag aagctagatc cagttgcagc aggatggccc ccgtgcttaa

3961 ggatcattgc ggcagtagcc ctaatggtga aggatgctga taaactcact tttgggcaac

4021 atctgaaggt agtgaccccc catgcgatcg agggggtcct gaaatatccc cctggtaggt

4081 ggatgactaa tgcccgacta acccattacc aaggactctt gctagatgca ccccggatca

4141 tcttcgctga acccaccgct ctgaatccag ccaccctgct gccgaccccg gatctgagag

4201 ctcccctgca tgattgccaa gagatcatgg cagaagtcac ccaggtgcgc cccgacctcc

4261 aggacaccgc actacccaac agtgagttgg tatggtacac tgatggaagc agcttcgtta

4321 tagatggtgt gcggagggca ggcgcagcgg tggtagacca agggggaaac atcatttgga

4381 atgcctcgct ttccccgggg acatcagcac agaaggccga actgatcgcg ctggcggagg

4441 cgctggaacg ggccaaaggg agacgagtga ctgtctacac cgatagccgc tacgcctttg

4501 gcactgtcca tgtgcatggc gctatctacc gggaaagagg ctttgttaca gcggaaggaa

4561 aggctctgcg caatcttcct gaggtacgaa gactgctgat ggctgtgcaa atgccccggg

4621 cagtcgcagt tgtccacatc cctgggcacc agtctgccca gaccccggaa gctgaaggaa

4681 accggcgagc ggatgaagcc gccaaggcag tggcagtagc ttcatcagct ttagcactca

4741 ccctgcccac acccgagctc cctcgcctgc ccccgcgacc tgactacact ccggaagacc

4801 tgcgatggat ccagaaccac cactgcccgg aatctgatca gcaggggtgg catcgggata

4861 cagaaagaag attgatactg ccggcacagc tagactgttt cttctctcca acctgcatca

4921 agccacccac ttaggaaaaa agaagttgct gacaattctc gagtccgccc gcctccggtt

4981 tccccgacaa gcggctcaga ttcaagagat tgtaaatcat ggcattgggt gccaggctat

5041 gagacccagt aggaaaggac cccaacatac aggtacgagg gtacggggaa gagcgccggg

5101 acggagttgg gaagtggatt ttactgaggt aaagcctggg aggtatgggt ataagtactt

5161 gctagtaatg gttgacacat tttcgggctg ggtggaagcc ttccccacga aacgggagac

5221 tgcccaagtg gttgctaagg cattactaga agaaattatt cccagatatg ggggttcctga

5281 ggttttaggc tccgataacg gcccagcttt catcagtaac gtcctacagg gactagcccg

5341 ggcgataggg atcaattgga agttacattg tgaatataat ccccagagct cagggcaggt

5401 agagagaatg aatcggactc taagaggaga cctttgtcca aactagccat cgagactggc

5461 ggggactggg tgaccctctt accctatgcc atcttccggg tccggaactc accatatgta

5521 catggtttga cacctttcga aattctgtat ggggcaccac cccccattat tgttcgtact

5581 ctaccagatc atgaccccaa tgtggcccca agttatctgg ccagtttaaa ggccctacaa

5641 ggggtccaac atgagatatg gcccctagtg agttccctgt atgaaattaa ggacgccccg

5701 aacccggaac atggcatcgt tccaggggat tgggtatggg taaggagaca caggtcccgg

5761 acactggagg aaagatggaa aggtccttat gtggttattc tggttacccc cactgcctta

5821 aaggttgacg gcattgggcc ttgggtccat cactctcacg tgcgccgagc cagccagctg

5881 gagaagacgc aagctaagga gtggatcgta cggcgacacc ctgataaccc tctgaagctg

5941 cagctctccc gacctcgggg aagcgtcaag ccccatgcct cagctaacga tggaatggct

6001 gctagcccta actctgctca acatctggga gaagacccac gcggggatca acccacacca

6061 accccaaaag ctaacatgga ccctaacaga tggacagacc caaacaaccc ttaatagcac

6121 cacacatact gcccccatca atacgtggtg gccagatttg tttttcgacc tgcgtgacat

6181 tttcggcact aaacgtggac ggcagtatga ctactcagtc aggtccaagc gggctgtaat

6241 tgacacctct caaggacata atgcacaagg gttttgggcc tgcccagggg acctaagaaa

6301 caattggaaa acctgtggcg gcccagaccg ctactattgg ggtagttgga gttgggtcac

6361 ctcctatgac gggccccgac agtgggacgt tgggaacagg gatctagtta aattctcctt

6421 tagggacccc cacaaccggg tgccccaggt atgtgtccag tttaaccaag acgtggcacg

6481 aagagagcgt ggttggttat caggattaac ttgggggttc caattagata taggccgttt

6541 ggcatggata ggcccccacc ccggcggtct cctaactatt cgactatcgg tggaaacgat

6601 cagcactcag gtgggtccaa ataaggtgct ggcccctctc gtccctacca agaacccagg

6661 tatatcaagg gataaaaaca ccgcaggagg gactgcggga acccacccaa agacctctgt

6721 aactccttcg acccctgcta cgcagacaac cgaagactca ttgcggaaac tggtgcgcac

6781 tgtatacgag acccttaatg ccaccagtcc taacctcaca acctcctgtt ggctgtgcta

6841 tgatgtaaag cccccgttct atgaggcaat aggacttaat gccacttacc acgcctctaa

6901 cgggaagaac ccttctcagt tttcatgggg gaatcataaa attggcttat ccatgcaact

6961 agtgagcggt catgttacct gtcaagggaa ggagccccag gctaaacaaa gtttatgtgc

7021 ctccatagac agctccccta gttggaaaag tgacactaag tggttaatcc ccagaactga

7081 tggatggtcg atatgttcaa cgactggctt caccccgtac ctatttacct tggtccttaa

7141 tgccgccaat gaattttgtg tcctagtaac agtgcagccc cgcatcctct atccccctga

7201 ggagagtatg tatttgcatt gggatagtga cacaagtatg agaagtaaaa gagagcccat

7261 caccgcagta accattgcca ccctgttcag tttgggaata gccggagccg ggaccggcat

7321 agcttccctg gctactcaac aatcaggaat gacctcccta agggcggcca tagatgagga

7381 catagaaagg ttggaaacct cgattagtca tttagagaag tcgctcacct ctctatccga

7441 ggtagtactc cagaatagaa gaggacttga tttgttgttc ctccaacaag ggggactgtg

7501 tgccgcgctg ggggaggaat gttgttttta tgctgaccat actggtgtgg taaaagaatc

7561 catggcaaag gtgagagaag ggttagctaa gagaaaacgg gaaagggaag ctcaggagaa

7621 ctggtttgag gcttggttta acagatcccc ctggctcacc accttagtat ctaccttagt

7681 gggcccaatt atcttgcttg tgcttattct aaccttgggc ccttgcatat taaacaagct

7741 tattaatttt gtaaaagatc gtgttaatac cgtccagctc atggtcctaa gacaacagta

7801 tgagacagtg cccacccgtg aggacctcta cggctggccc gtacatgagc aagattcctc

7861 attatgaaca acacacaggg gggaaatgta agagactgtt agcatgagaa ctacatcttt

7921 aggtcagggt taacagcccc tagcctgaaa cagcatagtg ggaaatgtaa gagactgtta

7981 gcatgagaac tacatcttta ggtcaggttt accagcccct agcctgaaac aacataatta

8041 taaaattcca ggatgtgggc agttacagca tggagacgag aagtcctgca gcttatctta

8101 tactcctggt ttccttgttc tgtagtaaat cttatactct ttgaagctat gcaagtcctg

8161 tagtttatct tatactcttt gaagctatgc aagtcctgta gtttatctag tactctctga

8221 agctatggaa aattactgag tgccttgaaa atgctatata acccctgggc tttaagtgtt

8281 gggggtcctg gtaaaaaccc gctgcgtcgg gcagagactc ggaccccagc tggctggaaa

8341 taaacctcgc tgtgtgactt gca

//

LOCUS OR761827 8363 bp RNA linear VRL 13-NOV-2023

DEFINITION Rhinolophus ferrumequinum retrovirus isolate Y6b.

ACCESSION OR761827

VERSION OR761827 GI:2619391290

KEYWORDS .

SOURCE Rhinolophus ferrumequinum retrovirus (RfRV)

ORGANISM Rhinolophus ferrumequinum retrovirus

Viruses; Riboviria; Pararnavirae; Artverviricota; Revtraviricetes;

Ortervirales; Retroviridae; Orthoretrovirinae; Gammaretrovirus; Bat

gammaretrovirus.

REFERENCE 1 (bases 1 to 8363)

AUTHORS Chung,H., Nguyen,V., Hwang,S., Chung,C. and Lee,J.

TITLE Identification of Rhinolophus ferrumequinum retrovirus of bats in

Korea

JOURNAL Unpublished

REFERENCE 2 (bases 1 to 8363)

AUTHORS Chung,H., Nguyen,V., Hwang,S., Chung,C. and Lee,J.

TITLE Direct Submission

JOURNAL Submitted (01-NOV-2023) Department of Microbiology and Immunology,

Institute for Immunology and Immunological Diseases, Yonsei

University College of Medicine, 50-1, Yonsei-ro, Seodaemun-gu,

Seoul Ks031, Korea

COMMENT ##Assembly-Data-START##

Assembly Method :: BioEdit v. 7.71

Sequencing Technology :: Illumina; Sanger dideoxy sequencing

##Assembly-Data-END##

FEATURES Location/Qualifiers

source 1..8363

/organism="Rhinolophus ferrumequinum retrovirus"

/mol_type="genomic RNA"

/isolate="Y6b"

/host="Rhinolophus ferrumequinum; bat"

/db_xref="taxon:1147756"

/country="South Korea"

/collection_date="2023-05-20"

CDS 621..2348

/codon_start=1

/product="gag protein"

/protein_id="WPA94631"

/db_xref="GI:-1675576005"

/translation="MGQVQVTPKTLLLNHFPEIRAKARNQGVEVKKGKFDPFCSAEWP

TFNVGWPPQGTFPLDIIKKVRDIINRRHPDQYPYILIWQALVESPPSWLKPFIPDKPE

GPPLPLKVLTVSGPSRQPAVPTAGPKPPEKPTQGPILQEGSDKYPSLIDLDLEETPPP

YAPVAPLQPRRAPSPVAPLLPEAAAPPELPHSTASPMAPPPPGSPAPAQGPARGLKPR

RRREETPEEEPSSSTSAGAPILPVRALGGTGPDGERAYQYWPFSSSDLYNWKAQNPPF

SEDPKGLTNLFESVMHTHSPTWDDCQQLLKTLFTTAERERIPTEARKNVPGDNGRPTT

LPNLIDERFPLNRLDWDFGNAEGRERLRVYRQTLMAGLRAAARRPTNLAKVKAIMQGE

NESPAVFLERLYDAYRQYTPLDPLAEENQSAVIMSFINQAAPDIRKKLYKQEGLGEMS

LRDLMKVAERVFNTRETPEKREDRIRKENQELQERIRKEDREHQSRENRRQQREMAKI

LLAGVQSTVRVGPSPAGPARPWRPRPRLDRGQCANCKEYGHWKRECPKRQGQTGQDAR

VLLAGMESD"

CDS <2349..4934

/note="pol protei"

/codon_start=1

/product="pol protein"

/protein_id="WPA94632"

/db_xref="GI:-1675576004"

/translation="GRRDSDPLPESWVTAYVEGKPVGFMVDTGAQYSVLNKPTEPLSQ

KTSLVQGATGSKAYRWTSKRQVDLGRHQVTHSFLVIPECPAPLLGRDLLTKNRAQIHF

EPDGIKLLDGQEQPLHILTLSLVDEHRLFALQDNPYNPPSTEWPRDMDYWLKTYPQAW

AEIAGVGRAARRAPVVVELKASARPIRIRQYPMSAEARKGIAPHINRLLEAGILKPCH

SAWNTPLLPVKKPGGKDYRPVQDLREVNKRVEDIHPTVPNPYTLLSHLPPSHVWYTTL

DLKDAFFSIALAPSSQHIFAFEWNNGNTGTPGQLTWTRLPQGFKNSPTLFNEALNQDL

DSFRQSHNSVTLLQYVDDLLLAAPSEAECRQATGDLLQELWQLGYRASAKKAQICRQT

VTYLGYKLKEGTRWLTEAMKETILRLPVPTSPQEVREFLGTTGYCRLWILGYAEIAKP

LYEATKDKVPWAWGSDQQKAYDELKVALLRAPALALPDPLKPFTLFVDERRGIAKGVL

MQRLGPWKRPVAYLSKKLDPVAAGWPPCLRIIAAVALMVKDADKLTFGQHLKVVTPHA

IEGVLKYPPGRWMTNARLTHYQGLLLDAPRIIFAEPTALNPATLLPTPDLRAPLHDCQ

EIMAEVTQVRPDLQDTALPNSELVWYTDGSSFVIDGVRRAGAAVVDQGGNIIWNASLS

PGTSAQKAELIALAEALERAKGRRVTVYTDSRYAFGTVHVHGAIYRERGFVTAEGKAL

RNLPEVRRLLMAVQMPRAVAVVHIPGHQSAQTPEAEGNRRADEAAKAVAVASSALALT

LPTPELPRLPPRPDYTPEDLRWIQNHHCPESDQQGWHRDTERRLILPAQLDCFFSPTC

IKPPT"

CDS 5990..7867

/codon_start=1

/product="envelope glycoprotein"

/protein_id="WPA94633"

/db_xref="GI:-1675576003"

/translation="MEWLLALTLLNIWEKTHAGINPHQPQKLTWTLTDGQTQTTLNST

THTAPINTWWPDLFFDLRDIFGTKRGRQYDYSVRSKRAVIDTSQGHNAQGFWACPGDL

RNNWKTCGGPDRYYWGSWSWVTSYDGPRQWDVGNRDLVKFSFRDPHNRVPQVCVQFNQ
DVARRERGWLSGLTWGFQLDIGRLAWIGPHPGGLLTIRLSVETISTQVGPNKVLAPLV
PTKNPGISRDKNTAGGTAGTHPKTSVTPSTPATQTTEDSLRKLVRTVYETLNATSPNL

TTSCWLCYDVKPPFYEAIGLNATYHASNGKNPSQFSWGNHKIGLSMQLVSGHVTCQGK
EPQAKQSLCASIDSSPSWKSDTKWLIPRTDGWSICSTTGFTPYLFTLVLNAANEFCVL
VTVQPRILYPPEESMYLHWDSDTSMRSKREPITAVTIATLFSLGIAGAGTGIASLATQ
QSGMTSLRAAIDEDIERLETSISHLEKSLTSLSEVVLQNRRGLDLLFLQQGGLCAALG

EECCFYADHTGVVKESMAKVREGLAKRKREREAQENWFEAWFNRSPWLTTLVSTLVGP
IILLVLILTLGPCILNKLINFVKDRVNTVQLMVLRQQYETVPTREDLYGWPVHEQDSS
L"

ORIGIN

1 cccttggctt taagtgtttg ggtccttgt taaaacccgc tgcgtcgggc agagactcgg

61 accccagctt gctggaaata aacctcgctg tgtgacttgc attattgtgt gggttctctg

121 tctgtctgag ggggacaatt ccggacctta acaggtgcgg caacgtccag gatgcccccc

181 cctcagggga acagacaact cccgataccg aggtttacat agggaaccgc ggaaggaggt

241 ttggccacct ccaatagggg aggactgaaa caggtcctgg accggagaga ctgaaacagg

301 tctctgcccg gtcccagttg agaattctca cgggaaggaa cgtgcgggtt acgaccctgg

361 aggctccgtt acttgaagtc gtgggagacg tccccgacga gaaggtggcc cagcgacgtc

421 cgcagtggac gccatttgga cgggccctgg gtaaggatct tgagcgtgtt gagactggtg

481 tgattgaaac ggcgcctgtg gatgtggtgt ggttgactgg ccggtgggtg ttgaagagtc

541 ccgtgcgaag ttgtgtattt gctggcactg ggtcttttgt cctttctttt gccttttctt

601 ggtttctctt tgtgacaatt atgggacagg ttcaggtaac acctaagacc ctgctcctga

661 accactttcc tgaaatccgc gccaaggctc gtaatcaagg tgtggaagtg aagaaaggta

721 agtttgatcc attctgctct gcagaatggc ctacttttaa tgtgggctgg cccccccagg

781 gaacttttcc cctagacatt attaagaagg tccgagatat tattaatcgg cgccatccgg

841 accaataccc ctatattttg atatggcaag ccttagtaga gagtcctccc tcctggctta

901 agccctttat ccccgacaag ccagaaggtc ccccctccc ccttaaagtc ctgaccgttt

961 cgggaccctc ccgccagccg gcggtgccca cggccggccc gaaacccccg gagaagccga

1021 cccagggacc catccttcag gaggggtcag acaaataccc ctccctgata gacctagatc

1081 tggaagagac cccccctcct tacgcgccgg tggcgccgct ccagccacgg cgcgcgccca

1141 gcccagtggc gccgcttttg cctgaggctg ccgcccctcc cgagctgcct cactctaccg

1201 cctccccgat ggctccccct cccccgggtt cccctgcccc agctcaaggg ccggcgaggg

1261 gattgaagcc tcgtagacgc cgggaagaga ccccagagga ggaaccgtcc tcctccacct

1321 ccgctggggc gccgattctc cccgtgcgag cactaggagg aactggtcca gatggggagc

1381 gagcatacca gtactggccc ttttctagca gtgatctgta caactggaag gctcaaaacc

1441 ctcctttttc tgaggacccg aaaggtctaa ctaacctgtt cgagtctgtc atgcacacac

1501 acagtcccac ttgggatgat tgccagcagc ttcttaagac cttattcacc accgcggagc

1561 gcgagcgaat ccccactgag gccagaaaga atgtccccgg cgacaacggg cggccgacga

1621 ccttgccgaa cctgatcgac gagcgctttc ccctgaatag actggattgg gactttggga

1681 acgcagaagg tagggagcgt ctccgagtct accgccagac tcttatggca ggtctccgag

1741 cggcggcacg ccgccccacc aatttggcca aggtaaaagc tataatgcaa ggggaaaatg

1801 aaagcccggc cgtgttttta gaacgcctct atgatgctta tagacagtac accccgttgg

1861 accccctcgc agaggaaaac cagtcggctg taattatgtc ctttataaac caggctgccc

1921 cagatattag gaagaaattg tacaaacagg agggactggg agaaatgtcc cttcgggatt

1981 taatgaaagt agcggagaga gtcttcaaca ctcgagagac tcccgaaaaa agggaggata

2041 gaattagaaa agaaaatcag gaattacagg aacgaatcag gaaggaagac agagagcatc

2101 agagcaggga aaacaggagg cagcagaggg agatggctaa gatcttgttg gcaggtgtgc

2161 aaagcacagt cagggtggga ccgagtccgg caggaccagc ccgaccgtgg agaccgcggc

2221 cccgactgga taggggacag tgtgcaaact gcaaggagta tggacattgg aagagggagt

2281 gccccaagcg ccagggccaa acagggcaag acgcacgggt cctgctggcg gggatggaga

2341 gtgactaggg gagacgggac tcggatcccc tccccgagtc ttgggtaact gcgtatgtgg

2401 aggggaagcc agtaggattc atggtagaca caggagccca gtactcagtt ttgaataagc

2461 ctacagagcc cttatctcag aaaaccagtt tggtacaagg ggcaactggg tccaaggctt

2521 atcggtggac tagtaagcgc caggtggact taggccgcca ccaagtgacc cactccttcc

2581 tagttatccc tgaatgccct gcccccttac tggggcgcga tctcctgact aagaacaggg

2641 cccagatcca ttttgagccg gatggcatta agctattgga tggccaagaa cagcccctcc

2701 acattttgac cctgtctctt gtggatgaac atcgcctgtt cgccctgcag gacaacccct

2761 acaaccctcc ctctacagaa tggccccgtg atatggatta ttggcttaaa acataccctc

2821 aggcgtgggc ggaaatagcg ggtgtgggcc gggcggcccg ccgagcacca gtagtggtgg

2881 aacttaaagc ctcggcccgg cctatccgga tccgccagta ccccatgtct gcagaggcgc

2941 ggaaagggat tgccccgcac attaaccgtt tactggaagc tggaatactg aaaccttgcc

3001 attctgcctg gaacacccca cttctccccg ttaagaaacc gggggaaaa gattataggc

3061 cagtccagga cttgagggaa gtgaataaga gggttgaaga catccacccc acggtcccca

3121 acccttatac cttactaagt cacttgcccc cttcacatgt ctggtatact accttagacc

3181 taaaggatgc gttttttagc atagccctgg cacccagcag ccaacacatt tttgccttcg

3241 aatggaataa tggcaatacg ggaacccccg ggcagctgac ctggactaga ctaccgcaag

3301 gcttcaaaaa ctctccaact ctgtttaatg aagccctaaa tcaggatttg gactcgtttc

3361 gccagagcca taattcagtt acgctcctgc agtacgtaga tgacttgctt ctggcggccc

3421 cctccgaagc tgaatgccga caggccactg gagacctcct ccaggagctg tggcagttgg

3481 gctatcgggc cagtgcaaag aaggctcaaa tatgcaggca aacagtcacc tacctggggt

3541 ataaactaaa agaaggaacc agatggctga cagaggccat gaaagagact attcttagac

3601 ttccagtccc gacctcacca caagaggtcc gtgaattttt agggacgaca ggctactgcc

3661 ggctgtggat tttggggtat gctgaaatag caaaacctct gtatgaggca accaaggata

3721 aggtcccttg ggcctggggg tcagaccaac aaaaggccta cgatgaactc aaggtcgctc

3781 tcctaagagc ccccggctctg gcattgccag accccctgaa gcccttcact ctctttgttg

3841 atgagaggag gggaatagcg aaaggggtgc taatgcagcg tctggggccc tggaaacgtc

3901 cggttgccta tttatccaag aagctagatc cagttgcagc aggatggccc ccgtgcttaa

3961 ggatcattgc ggcagtagcc ctaatggtga aggatgctga taaactcact tttgggcaac

4021 atctgaaggt agtgaccccc catgcgatcg agggggtcct gaaatatccc cctggtaggt

4081 ggatgactaa tgcccgacta acccattacc aaggactctt gctagatgca ccccggatca

4141 tcttcgctga acccaccgct ctgaatccag ccaccctgct gccgaccccg gatctgagag

4201 ctcccctgca tgattgccaa gagatcatgg cagaagtcac ccaggtgcgc cccgacctcc

4261 aggacaccgc actacccaac agtgagttgg tatggtacac tgatggaagc agcttcgtta

4321 tagatggtgt gcggagggca ggcgcagcgg tggtagacca agggggaaac atcatttgga

4381 atgcctcgct ttccccgggg acatcagcac agaaggccga actgatcgcg ctggcggagg

4441 cgctggaacg ggccaaaggg agacgagtga ctgtctacac cgatagccgc tacgcctttg

4501 gcactgtcca tgtgcatggc gctatctacc gggaaagagg ctttgttaca gcggaaggaa

4561 aggctctgcg caatcttcct gaggtacgaa gactgctgat ggctgtgcaa atgccccggg

4621 cagtcgcagt tgtccacatc cctgggcacc agtctgccca gaccccggaa gctgaaggaa

4681 accggcgagc ggatgaagcc gccaaggcag tggcagtagc ttcatcagct ttagcactca

4741 ccctgcccac acccgagctc cctcgcctgc ccccgcgacc tgactacact ccggaagacc

4801 tgcgatggat ccagaaccac cactgcccgg aatctgatca gcaggggtgg catcgggata

4861 cagaaagaag attgatactg ccggcacagc tagactgttt cttctctcca acctgcatca

4921 agccacccac ttaggaaaaa agaagttgct gacaattctc gagtccgccc gcctccggtt

4981 tccccgacaa gcggctcaga ttcaagagat tgtaaatcat ggcattgggt gccaggctat

5041 gagacccagt aggaaaggac cccaacatac aggtacgagg gtacggggaa gagcgccggg

5101 acggagttgg gaagtggatt ttactgaggt aaagcctggg aggtatgggt ataagtactt

5161 gctagtaatg gttgacacat tttcgggctg ggtggaagcc ttccccacga aacgggagac

5221 tgcccaagtg gttgctaagg cattactaga agaaattatt cccagatatg ggttcctga

5281 ggttttaggc tccgataacg gcccagcttt catcagtaac gtcctacagg gactagcccg

5341 ggcgataggg atcaattgga agttacattg tgaatataat ccccagagct cagggcaggt

5401 agagagaatg aatcggactc taagaggaga cctttgtcca aactagccat cgagactggc

5461 ggggactggg tgaccctctt accctatgcc atcttccggg tccggaactc accatatgta

5521 catggtttga cacctttcga aattctgtat ggggcaccac cccccattat tgttcgtact

5581 ctaccagatc atgaccccaa tgtggcccca agttatctgg ccagtttaaa ggccctacaa

5641 ggggtccaac atgagatatg gcccctagtg agttccctgt atgaaattaa ggacgccccg

5701 aacccggaac atggcatcgt tccaggggat tgggtatggg taaggagaca caggtcccgg

5761 acactggagg aaagatggaa aggtccttat gtggttattc tggttacccc cactgcctta

5821 aaggttgacg gcattgggcc ttgggtccat cactctcacg tgcgccgagc cagccagctg

5881 gagaagacgc aagctaagga gtggatcgta cggcgacacc ctgataaccc tctgaagctg

5941 cagctctccc gacctcgggg aagcgtcaag ccccatgcct cagctaacga tggaatggct

6001 gctagcccta actctgctca acatctggga gaagacccac gcggggatca acccacacca

6061 accccaaaag ctaacatgga ccctaacaga tggacagacc caaacaaccc ttaatagcac

6121 cacacatact gcccccatca atacgtggtg gccagatttg tttttcgacc tgcgtgacat

6181 tttcggcact aaacgtggac ggcagtatga ctactcagtc aggtccaagc gggctgtaat

6241 tgacacctct caaggacata atgcacaagg gttttgggcc tgcccagggg acctaagaaa

6301 caattggaaa acctgtggcg gcccagaccg ctactattgg ggtagttgga gttgggtcac

6361 ctcctatgac gggccccgac agtgggacgt tgggaacagg gatctagtta aattctcctt

6421 tagggacccc cacaaccggg tgccccaggt atgtgtccag tttaaccaag acgtggcacg

6481 aagagagcgt ggttggttat caggattaac ttgggggttc caattagata taggccgttt

6541 ggcatggata ggcccccacc ccggcggtct cctaactatt cgactatcgg tggaaacgat

6601 cagcactcag gtgggtccaa ataaggtgct ggcccctctc gtccctacca agaacccagg

6661 tatatcaagg gataaaaaca ccgcaggagg gactgcggga acccacccaa agacctctgt

6721 aactccttcg acccctgcta cgcagacaac cgaagactca ttgcggaaac tggtgcgcac

6781 tgtatacgag acccttaatg ccaccagtcc taacctcaca acctcctgtt ggctgtgcta

6841 tgatgtaaag cccccgttct atgaggcaat aggacttaat gccacttacc acgcctctaa

6901 cgggaagaac ccttctcagt tttcatgggg gaatcataaa attggcttat ccatgcaact

6961 agtgagcggt catgttacct gtcaagggaa ggagccccag gctaaacaaa gtttatgtgc

7021 ctccatagac agctccccta gttggaaaag tgacactaag tggttaatcc ccagaactga

7081 tggatggtcg atatgttcaa cgactggctt caccccgtac ctatttacct tggtccttaa

7141 tgccgccaat gaattttgtg tcctagtaac agtgcagccc cgcatcctct atccccctga

7201 ggagagtatg tatttgcatt gggatagtga cacaagtatg agaagtaaaa gagagcccat

7261 caccgcagta accattgcca ccctgttcag tttgggaata gccggagccg ggaccggcat

7321 agcttccctg gctactcaac aatcaggaat gacctcccta agggcggcca tagatgagga

7381 catagaaagg ttggaaacct cgattagtca tttagagaag tcgctcacct ctctatccga

7441 ggtagtactc cagaatagaa gaggacttga tttgttgttc ctccaacaag ggggactgtg

7501 tgccgcgctg ggggaggaat gttgtttta tgctgaccat actggtgtgg taaaagaatc

7561 catggcaaag gtgagagaag ggttagctaa gagaaaacgg gaaagggaag cccaggagaa

7621 ctggtttgag gcttggttta acagatcccc ctggctcacc accttagtat ctaccttagt

7681 gggcccaatt atcttgcttg tgcttattct aaccttgggc ccttgcatat taaacaagct

7741 tattaatttt gtaaaagatc gtgttaatac cgtccagctc atggtcctaa ggcaacagta

7801 tgagacagtg cccacccgtg aggacctcta cggctggccc gtacatgagc aagattcctc

7861 attatgaaca acacacaggg gggaaatgta agagactgtt agcatgagaa ctacatcttt

7921 aggtcagggt taacagcccc tagcctgaaa cagcatagtg ggaaatgtaa gagactgtta

7981 gcatgagaac tacatcttta ggtcaggttt accagcccct agcctgaaac aacataatta

8041 taaaattcca ggatgtgggc agttacagca tggagacgag aagtcctgca gcttatctta

8101 tactcctggt ttccttgttc tgtagtaaat cttatactct ttgaagctat gcaagtcctg

8161 tagtttatct tatactcttt gaagctatgc aagtcctgta gtttatctag tactctctga

8221 agctatggaa aattactgag tgccttgaaa atgctatata acccctgggc tttaagtgtt

8281 gggggtcctg gtaaaaaccc gctgcgtcgg gcagagactc ggaccccagc tggctggaaa

8341 taaacctcgc tgtgtgactt gca

//

LOCUS OR761828 8363 bp RNA linear VRL 13-NOV-2023

DEFINITION Rhinolophus ferrumequinum retrovirus isolate Y7a.

ACCESSION OR761828

VERSION OR761828 GI:2619391294

KEYWORDS .

SOURCE Rhinolophus ferrumequinum retrovirus (RfRV)

ORGANISM Rhinolophus ferrumequinum retrovirus

Viruses; Riboviria; Pararnavirae; Artverviricota; Revtraviricetes;

Ortervirales; Retroviridae; Orthoretrovirinae; Gammaretrovirus; Bat

gammaretrovirus.

REFERENCE 1 (bases 1 to 8363)

AUTHORS Chung,H., Nguyen,V., Hwang,S., Chung,C. and Lee,J.

TITLE Identification of Rhinolophus ferrumequinum retrovirus of bats in

Korea

JOURNAL Unpublished

REFERENCE 2 (bases 1 to 8363)

AUTHORS Chung,H., Nguyen,V., Hwang,S., Chung,C. and Lee,J.

TITLE Direct Submission

JOURNAL Submitted (01-NOV-2023) Department of Microbiology and Immunology,

Institute for Immunology and Immunological Diseases, Yonsei

University College of Medicine, 50-1, Yonsei-ro, Seodaemun-gu,

Seoul Ks031, Korea

COMMENT ##Assembly-Data-START##

Assembly Method :: BioEdit v. v. 7.71

Sequencing Technology :: Illumina; Sanger dideoxy sequencing

##Assembly-Data-END##

FEATURES Location/Qualifiers

source 1..8363

/organism="Rhinolophus ferrumequinum retrovirus"

/mol_type="genomic RNA"

/isolate="Y7a"

/host="Rhinolophus ferrumequinum; bat"

/db_xref="taxon:1147756"

/country="South Korea"

/collection_date="2023-05-20"

CDS 621..2348

/codon_start=1

/product="gag protein"

/protein_id="WPA94634"

/db_xref="GI:-1675576001"

/translation="MGQVQVTPKTLLLNHFPEIRAKARNHGVEVKKGKFDTFCSAEWP

TFNVGWPPQGTFPLDIIKKVRDLINRRHPDQYPYILIWQALVESPPSWLKPFIPDKPE

GPPLPLKVLTVSGPSRQPAVPTAGPKPPEKPTQGPILQEGSDKYPSLIDLDLEETPPP

YAPVAPLQPRRAPSPVAPLLPEAAAPPELPHSTASPMAPPPPGSPAPAQGPARGLKPR

RRREETPEEEPSSSTSAGAPILPVRALGGTGPDGERAYQYWPFSSSDLYNWKAQNPPF

SEDPKGLTNLFESVMHTHSPTWDDCQQLLKTLFTTEERERILTEARKKVPGDNGRPTT

LPNLIDERFPLNRLDWDFGNAEGRERLRVYRQTLMAGLRAAARRPTNLAKVKAIMQGE

NESPAVFLERLYDAYRQYTPLDPLAEENQSAVIMSFINQAAPDIRKKLYKQEGLGEMS

LRDLMKVAERVFNTRETPEKREDRIRKENQELQERIRKEDREHQSRENRRQQREMAKI

LLAGVQSTVRVGPSPAGPARPWRPRPRLDRGQCANCKEYGHWKRECPKRQGQTGQDAR

VLLAGMESD"

CDS <2349..4934

/codon_start=1

/product="pol protein"

/protein_id="WPA94635"

/db_xref="GI:-1675576000"

/translation="GRRDSDPLPESWVTAYVEGKPVGFMVDTGAQYSVLNKPTEPLSQ

KTSLVQGATGSKAYRWTSKRQVDLGRHQVTHSFLVIPECPAPLLGRDLLTKIRAQIHF

EPDGIKLLDGQEQPLHILTLSLVDEHRLFALQDNPYNPPSTEWPRDMDYWLKTYPQAW

AEIAGVGRAARRAPVVVELKASARPIRIRQYPMSAEARKGNAPHINRLLEAGILKPCH

SAWNTPLLPVKKPGGKDYRPVQDLREVNKRVEDIHPTVPNPYTLLSHLPPSHVWYTTL

DLKDAFFSIALAPSNQHIFAFEWNNGNTGTPGQLTWTRLPQGFKNSPTLFNEALNQDL

DSFRQSHNSVTLLQYVDDLLLAAPSEAECRQATGDLRQELGQLGYRASAKKAQICRQT

VTYLGYKLKEGTRWLTEAMKETILRLPVPTSPQEVREFLGTTGYCRLWILGYAEIAKP

LYEATKDKVPWAWGSDQQKAYDELKVALLRAPALALPDPLKPFTLFVDERRGIAKGVL

MQRLGPWKRPVAYLSKKLDPVAAGWPPCLRIIGAVALMVKDADKLTFGQHLKVVTPHA

IEGVLKYPPGRWMTNARLTHYQGLLLDAPRIIFAEPTALNPATLLPTPDLRAPLHDCQ

EIMAEVTQVRPDLQDTALPNSELVWYTDGSSFVIDGVRRAGAAVVDQGGNIIWNASLS

PGTSAQKAELIALAEALERAKGRRVTVYTDSRYAFGTVHVHGAIYRERGFVTAEGKAL

RNLPEVRRLLMAVQMPRAVAVVHIPGHQSAQTPEAEGNRRADEAAKAVAVASSALALT

LPTPELPRLPPRPDYTPEDLRWIQNHHCPESDQQGWHRDTERRLILPAQLDCFFSPTC

IKPPT"

CDS 5990..7867

/codon_start=1

/product="envelope glycoprotein"

/protein_id="WPA94636"

/db_xref="GI:-1675575999"

/translation="MEWLLALTLLNIWEKSHAGINPHQPQKLTWTLTDGQTQTTLNST

TNTAPINTWWPDLFFDLRDIFGTKRGRQYDYSVRSKRAVIDTSQGHSAQGFWACPGNL

RNNWKTCGGPDRYYWGSWSWVTSYDGPRQWDVGNRDLVKFSFRDPHNRVPQVCVQFNQ

DVARRERGWLSGLTWGFQLDIGRLAWIGPHPGGLLTIRLSVETISTQVGPNKVLAPLV

PTKNPGISRDKNTAGGTAGTHPKTSVTPSTPATQTTEDSLRKLVRTVYETLNATSPNL

TTSCWLCYDVKPPFYEAIGLNATYHASNGKNPSQFSWGNHKIGLSMQLVSGHVTCQGK

EPQAKQSLCASIDSSPSWKSDTKWLIPRTDGWSICSTTGFTPYLFTLVLNAANEFCVL

VTVQPRILYPPEESMYLHWDSATSMRSKREPITAVTIATLFSLGIAGAGTGIASLATQ

QSGMTSLRAAIDEDIERLETSISHLEKSLTSLSEVVLQNRRGLDLLFLQQGGLCAALG

EECCFYADHTGVVKESMAKVREGLAKRKREREAQENWFEAWFNRSPWLTTLVSTLVGP

IILLVLILTLGPCILNKLINFVKDRVNTVQLMVLRQQYETVPTREDLYGWPVHEQDSS

L"

ORIGIN

1 cccttggctt taagtgtttg gggtccttgt taaaacccgc tgcgtcgggc agagactcgg

61 accccagctt gctggaaata aacctcgctg tgtgacttgc attattgtgt gggttctctg

121 tctgtctgag ggggacaatt ccggacctta acaggtgcgg caacgtccag gatgcccccc

181 cctcagggga acagacaact cccgataccg aggtttacat agggaaccgc ggaaggaggt

241 ttggccacct ccaatagggg aggactgaaa caggtcctgg accggagaga ctgaaacagg

301 tctctgcccg gtcccagttg agaattctca cgggaaggaa cgtgcgggtt acgaccctgg

361 aggctccgtt acttgaagtc gtgggagacg tccccgacga gaaggtggcc cagcgacgtc

421 cgcagtggac gccatttgga cgggccctgg gtaaggatct tgagcgtgtt gagactggtg

481 tgattgaaac ggcgcctgtg gatgtggtgt ggttgactgg ccggtgggtg ttgaagagtc

541 ccgtgcgaag ttgtgtattt gctggcactg ggtcttttgt cctttctttt gccttttctt

601 ggtttctctt tgtgacaatt atgggacagg ttcaggtaac acctaagacc ctgctcctga

661 accactttcc tgaaatccgc gccaaggctc gtaatcatgg tgtggaagtg aagaaaggta

721 agtttgatac attctgctct gcagaatggc ctacttttaa tgtgggctgg cccccccagg

781 gaacttttcc cctagacatt attaagaagg tccgagatct tattaatcgg cgccatccgg

841 accaataccc ctatattttg atatggcaag ccttagtaga gagtcctccc tcctggctta

901 agccctttat ccccgacaag ccagaaggtc ccccctccc ccttaaagtc ctgaccgttt

961 cgggaccctc ccgccagccg gcggtgccca cggccggccc gaaacccccg gagaagccga

1021 cccagggacc catccttcag gaggggtcag acaaataccc ctccctgata gacctagatc

1081 tggaagagac cccccctcct tacgcgccgg tggcgccgct ccagccacgg cgcgcgccca

1141 gcccagtggc gccgcttttg cctgaggctg ccgcccctcc cgagctgcct cactctaccg

1201 cctccccgat ggctccccct cccccgggtt cccctgcccc agctcaaggg ccggcgaggg

1261 gattgaagcc tcgtagacgc cgggaagaga ccccagagga ggaaccgtcc tcctccacct

1321 ccgctggggc gccgattctc cccgtgcgag cactaggagg aactggtcca gatggggagc

1381 gagcatacca gtactggccc ttttctagca gtgatctgta caactggaag gctcaaaacc

1441 ctcctttttc tgaggacccg aaaggtctaa ctaacctgtt cgagtctgtc atgcacacac

1501 acagtcccac ttgggatgat tgccagcagc ttcttaagac cttattcacc accgaggagc

1561 gcgagcgaat cctcactgag gccagaaaga aagtccccgg cgacaacggg cggccgacga

1621 ccttgccgaa cctgatcgac gagcgctttc ccctgaatag actggattgg gactttggga

1681 acgcagaagg tagggagcgt ctccgagtct accgccagac tcttatggca ggtctccgag

1741 cggcggcacg ccgccccacc aatttggcca aggtaaaagc tataatgcaa ggggaaaatg

1801 aaagcccggc cgtgttttta gaacgcctct atgatgctta taggcagtac accccgttgg

1861 accccctcgc agaggaaaac cagtcggctg taattatgtc ctttataaac caggctgccc

1921 cagatattag gaagaaattg tacaaacagg agggactggg agaaatgtcc cttcgggatt

1981 taatgaaagt agcggagaga gtcttcaaca ctcgagagac tcccgaaaaa agggaggata

2041 gaattagaaa agaaaatcag gaattacagg aacgaatcag gaaggaagac agagagcatc

2101 agagcaggga aaacaggagg cagcagaggg agatggctaa gatcttgttg gcaggtgtgc

2161 aaagcacagt cagggtggga ccgagtccgg caggaccagc ccgaccgtgg agaccgcggc

2221 cccgactgga taggggacag tgtgcaaact gcaaggagta tggacattgg aagagggagt

2281 gccccaagcg ccagggccaa acagggcaag acgcacgggt cctgctggcg ggatggaga

2341 gtgactaggg gagacgggac tcggatcccc tccccgagtc ttgggtaact gcgtatgtgg

2401 aggggaagcc agtaggattc atggtagaca caggagccca gtactcagtt ttgaataagc

2461 ctacagagcc cttatctcag aaaaccagtt tggtacaagg ggcaactggg tccaaggctt

2521 atcggtggac tagtaagcgc caggtggact taggccgcca ccaagtgacc cactccttcc

2581 tagttatccc tgaatgccct gcccccttac tggggcgcga tctcctgact aagatcaggg

2641 ctcagatcca ttttgagccg gatggcatta agctattgga tggccaagaa cagcccctcc

2701 acattttgac cctgtctctt gtggatgaac atcgcctgtt cgccctgcag gacaacccct

2761 acaaccctcc ctctacagaa tggccccgtg atatggatta ttggcttaaa acataccctc

2821 aggcgtgggc ggaaatagcg ggtgtgggcc gggcggcccg ccgagcacca gtagtggtgg

2881 aacttaaagc ctcggcccgg cctatccgga tccgccagta ccccatgtct gcagaggcgc

2941 ggaaagggaa tgccccgcac attaaccgtt tactggaagc tggaatactg aaaccttgcc

3001 attctgcctg gaacacccca cttctccccg ttaagaaacc ggggggaaaa gattataggc

3061 cagtccagga cttgagggaa gtgaataaga gggttgaaga catccacccc acggtcccca

3121 acccttatac cttactaagt cacttgcccc cttcacatgt ctggtatact accttagacc

3181 taaaggatgc gttttttagc atagccctgg cacccagcaa ccaacacatt tttgccttcg

3241 aatggaataa tggcaatacg ggaacccccg ggcagctgac ctggactaga ctaccgcaag

3301 gcttcaaaaa ctctccaact ctgtttaatg aagccctaaa tcaggatttg gactcgtttc

3361 gccagagcca taattcagtt acgctcctgc agtacgtaga tgacttgctt ctggcggccc

3421 cctccgaagc tgaatgccga caggccactg gagacctccg ccaggagctg ggcagttgg

3481 gctatcgggc cagtgcaaag aaggctcaaa tatgcaggca aacagtcacc tacctggggt

3541 ataaactaaa agaaggaacc agatggctga cagaggccat gaaagagact attcttagac

3601 ttccagtccc gacctcacca caagaggtcc gtgaattttt agggacgaca ggctactgcc

3661 ggctgtggat tttggggtat gctgaaatag caaaacctct gtatgaggca accaaggata

3721 aggtcccttg ggcctggggg tcagaccaac aaaaggccta cgatgaactc aaggtcgctc

3781 tcctaagagc ccggctctg gcattgccag accccctgaa gcccttcact ctctttgttg

3841 atgagaggag gggaatagcg aaaggggtgc taatgcagcg tctggggccc tggaaacgtc

3901 cggttgccta tttatccaag aagctagatc cagttgcagc aggatggccc ccgtgcttaa

3961 ggatcattgg ggcagtagcc ctaatggtga aggatgctga taaactcact tttgggcaac

4021 atctgaaggt agtgaccccc catgcgatcg agggggtcct gaaatatccc cctggtaggt

4081 ggatgactaa tgcccgacta acccattacc aaggactctt gctagatgca ccccggatca

4141 tcttcgctga acccaccgct ctgaatccag ccaccctgct gccgaccccg gatctgagag

4201 ctcccctgca tgattgccaa gagatcatgg cagaagtcac ccaggtgcgc cccgacctcc

4261 aggacaccgc actacccaac agtgagttgg tatggtacac tgatggaagc agcttcgtta

4321 tagatggtgt gcggagggca ggcgcagcgg tggtagacca agggggaaac atcatttgga

4381 atgcctcgct ttccccgggg acatcagcac agaaggccga actgatcgcg ctggcggagg

4441 cgctggaacg ggccaaaggg agacgagtga ctgtctacac cgatagccgc tacgcctttg

4501 gcactgtcca tgtgcatggc gctatctacc gggaaagagg ctttgttaca gcggaaggaa

4561 aggctctgcg caatcttcct gaggtacgaa gactgctgat ggctgtgcaa atgccccggg

4621 cagtcgcagt tgtccacatc cctgggcacc agtctgccca gaccccggaa gctgaaggaa

4681 accggcgagc ggatgaagcc gccaaggcag tggcagtagc ttcatcagct ttagcactca

4741 ccctgcccac acccgagctc cctcgcctgc ccccgcgacc tgactacact ccggaagacc

4801 tgcgatggat ccagaaccac cactgcccgg aatctgatca gcaggggtgg catcgggata

4861 cagaaagaag attgatactg ccggcacagc tagactgttt cttctctcca acctgcatca

4921 agccacccac ttaggaaaaa agaagttgct gacaattctc gagtccgccc gcctccggtt

4981 tccccgacaa gcggctcaga ttcaagagat tgtaaatcat ggcattgggt gccaggctat

5041 gagacccagt aggaaaggac cccaacatac aggtacgagg gtacggggaa gagcgccggg

5101 acggagttgg gaagtggatt ttactgaggt aaagcctggg aggtatgggt ataagtactt

5161 gctagtaatg gttgacacat tttcgggctg ggtggaagcc ttccccacga aacgggagac

5221 tgcccaagtg gttgctaagg cattactaga agaaattatt cccagatatg gggttcctga

5281 ggttttaggc tccgataacg gcccagcttt catcagtaac gtcctacagg gactagcccg

5341 ggcgataggg atcaattgga agttacattg tgaatataat ccccagagct cagggcaggt

5401 agagagaatg aatcggactc taagaggaga cctttgtcca aactagccat cgagactggc

5461 ggggactggg tgaccctctt accctatgcc atcttccggg tccggaactc accatatgta

5521 catggtttga cacctttcga aattctgtat ggggcaccac cccccattat tgttcgtact

5581 ctaccagatc atgaccccaa tgtggcccca agttatctgg ccagtttaaa ggccctacaa

5641 ggggtccaac atgagatatg gcccctagtg agttccctgt atgaaattaa ggacgccccg

5701 aacccggaac atggcatcgt tccaggggat tgggtatggg taaggagaca caggtcccgg

5761 acactggagg aaagatggaa aggtccttat gtggttattc tggttacccc cactgcctta

5821 aaggttgacg gcattgggcc ttgggtccat cactctcacg tgcgccgagc cagccagctg

5881 gagaagacgc aagctaagga gtggatcgta cggcgacacc ctgataaccc tctgaagctg

5941 cagctctccc gacctcgggg aagcgtcaag ccccatgcct cagctaacga tggaatggct

6001 gctagcccta actctgctca acatctggga gaagagccac gcggggatca acccacacca

6061 accccaaaag ctaacatgga ccctaacaga tggacagacc caaacaaccc ttaatagcac

6121 cacaaatact gcccccatca atacgtggtg gccagatttg tttttcgacc tgcgtgacat

6181 tttcggcact aaacgtggac ggcagtatga ctactcagtc aggtccaagc gggctgtaat

6241 tgacacctct caaggacata gtgcacaagg gttttgggcc tgcccaggga acctaagaaa

6301 caattggaaa acctgtggcg gcccagaccg ctactattgg ggtagttgga gttgggtcac

6361 ctcctatgac gggccccgac agtgggacgt tgggaacagg gatctagtta aattctcctt

6421 tagggacccc cacaaccggg tgccccaggt atgtgtccag tttaaccaag acgtggcacg

6481 aagagagcgt ggttggttat caggattaac ttgggggttc caattagata taggccgttt

6541 ggcatggata ggcccccacc ccggcggtct cctaactatt cgactatcgg tggaaacgat

6601 cagcactcag gtgggtccaa ataaggtgct ggcccctctc gtccctacca agaacccagg

6661 tatatcaagg gataaaaaca ccgcaggagg gactgcggga acccacccaa agacctctgt

6721 aactccttcg acccctgcta cgcagacaac cgaagactca ttgcggaaac tggtgcgcac

6781 tgtatacgag acccttaatg ccaccagtcc taacctcaca acctcctgtt ggctgtgcta

6841 tgatgtaaag cccccgttct atgaggcaat aggacttaat gccacttacc acgcctctaa

6901 cgggaagaac ccttctcagt tttcatgggg gaatcataaa attggcttat ccatgcaact

6961 agtgagcggt catgttacct gtcaagggaa ggagccccag gctaaacaaa gtttatgtgc

7021 ctccatagac agctccccta gttggaaaag tgacactaag tggttaatcc ccagaactga

7081 tggatggtcg atatgttcaa cgactggctt caccccgtac ctatttacct tggtccttaa

7141 tgccgccaat gaattttgtg tcctagtaac agtgcagccc cgcatcctct atccccctga

7201 ggagagtatg tatttgcatt gggatagtgc cacaagtatg agaagtaaaa gagagcccat

7261 caccgcagta accattgcca ccctgttcag tttgggaata gccggagccg ggaccggcat

7321 agcttccctg gctactcaac aatcaggaat gacctcccta agggcggcca tagatgagga

7381 catagaaagg ttggaaacct cgattagtca tttagagaag tcgctcacct ctctatccga

7441 ggtagtactc cagaatagaa gaggacttga tttgttgttc ctccaacaag ggggactgtg

7501 tgccgcgctg ggggaggaat gttgttttta tgctgaccat actggtgtgg taaaagaatc

7561 catggcaaag gtgagagaag ggttagctaa gagaaaacgg gaaagggaag ctcaggagaa

7621 ctggtttgag gcttggttta acagatcccc ctggctcacc accttagtat ctaccttagt

7681 gggcccaatt atcttgcttg tgcttattct aaccttgggc ccttgcatat taaacaagct

7741 tattaatttt gtaaaagatc gtgttaatac cgtccagctc atggtcctaa gacaacagta

7801 tgagacagtg cccacccgtg aggacctcta cggctggccc gtacatgagc aagattcctc

7861 attatgaaca acacacaggg gggaaatgta agagactgtt agcatgagaa ctacatcttt

7921 aggtcagggt taacagcccc tagcctgaaa cagcatagtg ggaaatgtaa gagactgtta

7981 gcatgagaac tacatcttta ggtcaggttt accagcccct agcctgaaac aacataatta

8041 taaaattcca ggatgtgggc agttacagca tggagacgag aagtcctgca gcttatctta

8101 tactcctggt ttccttgttc tgtagtaaat cttatactct ttgaagctat gcaagtcctg

8161 tagtttatct tatactcttt gaagctatgc aagtcctgta gtttatctag tactctctga

8221 agctatggaa aattactgag tgccttgaaa atgctatata acccctgggc tttaagtgtt

8281 gggggtcctg gtaaaaaccc gctgcgtcgg gcagagactc ggaccccagc tggctggaaa

8341 taaacctcgc tgtgtgactt gca

//
