# Supplementary material for: Identification and characterization of recent retrovirus in Rhinolophus ferrumequinum bats
Source: Microbiol Spectr. 2024 Apr 30;12(6):e04323-23. doi: 10.1128/spectrum.04323-23 (PMC11237596; doi:10.1128/spectrum.04323-23)
Supplement: Supplemental Table 1 — List of primers used in this study. [file spectrum.04323-23-s0004.pdf]

Supplementary Table 1. List of primers used in this study.

Primer names	Detection method	Sequence (5'-3')	Location (nt)	Purpose	Size (bp)
RfRV-F	PCR	TACCCTATGCCATCTTCCGG	5480-5913	Detection	434
RfRV-R		CCGTACGATCCACTCCTCAG			
Bat-polF	Digital PCR	AGACTACCGCAAGGCTTCAA	3288-3374	Quantification	87
Bat-polR		ACTGCAGGAGCGTAACTGAA			
Bat-pol Probe		Fam-TGGACTCGTTTCGCCAGAGCCA-BHQ1			
1F	PCR	CCCTTGGCTTTAAGTGTTTGGG	1-1354	Genome Sequencing	1354
1R		TAGTGCTCGCACGGGGAGAA			
2F	PCR	GAGGGGATTGAGGCCTCGTA	1256-2632	Genome Sequencing	1377
2R		CTTAGTCAGGAGATCGCGCC			
3F	PCR	TGGACTTAGGCCGCCACCAA	2545-3947	Genome Sequencing	1403
3R		GCCATCCTGCTGCAACTGGA			
4F	PCR	GGAGGGGAATAGCGAAAGGG	3847-5240	Genome Sequencing	1394
4R		GCCTTAGCAACCACTTGGGC			
5F	PCR	ACCCCAACATACAGGTACGA	5059-6585	Genome Sequencing	1527
5R		AATAGTTAGGAGACCGCCGG			
6F	PCR	CCCCAGGTACGTGTCCAGTT	6443-7852	Genome Sequencing	1410
6R		CTTGCTCATGTACGGGCCAG			
7F	PCR	TACCGTCCAGCTCATGGTCC	7768-8363	Genome Sequencing	596
7R		TGCAAGTCACACAGCGAGGTTTAT			
