# Supplementary material for: Identification and characterization of recent retrovirus in Rhinolophus ferrumequinum bats
Source: Microbiol Spectr. 2024 Apr 30;12(6):e04323-23. doi: 10.1128/spectrum.04323-23 (PMC11237596; doi:10.1128/spectrum.04323-23)
Supplement: Supplemental Table 2 — This sequences used in tree analysis. [file spectrum.04323-23-s0005.pdf]

Supplementary Table 2. This sequences used in tree analysis

Number	GenBank number	Isolation name	Host	Reference
1	AB750369	HIV-1	Human	Cui, et al
2	AF033816	HSRV	Human	Cui, et al
3	AF151794	KoRV	Koala	Cui, et al
4	AJ000387	Gypsy	Insect	Cui, et al
5	AJ293656	PERV A	Pig	Cui, et al
6	AJ506107	Xen1	Amphibians	Cui, et al
7	AY099324	PERV B	Pig	Cui, et al
8	D10032	BaEV	Monkey	Cui, et al
9	D11078	HERV H/RGH2	Human	Cui, et al
10	DQ112147	HERV K	Human	Cui, et al
11	EF133960	PERV C	Pig	Cui, et al
12	GQ222416	OOEV	Whale	Cui, et al
13	JQ303225	RfRV	Bat	Cui, et al
14	KP890355	HIV-2	Human	Cui, et al
15	M16575	EIAV	Equine	Cui, et al
16	M80216	JSRV	Sheep	Cui, et al
17	NC001362	F-MuLV FB29	Mouse	Cui, et al
18	NC001407	RSV	Avian	Cui, et al
19	NC001408	ALV	Avian	Cui, et al
20	NC001414	BLV	Bovine	Cui, et al
21	NC001436	HTLV 1	Human	Cui, et al
22	NC001488	HTLV 2	Human	Cui, et al
23	NC001501	M-MuLV	Mouse	Cui, et al
24	NC001503	MMTV	Mouse	Cui, et al
25	NC001550	MPMV	Monkey	Cui, et al
26	NC001702	M-CRV	Mouse	Cui, et al
27	NC001724	SnRV	Snake	Cui, et al
28	NC001819	R-MuLV	Mouse	Cui, et al
29	NC001867	WDSV	Fish	Cui, et al
30	NC001885	GALV	Gibbon	Cui, et al
31	NC001940	FeLV FRA	Cat	Cui, et al
32	NC007815	MuLV 1,2	Mouse	Cui, et al
33	NC009889	RD114	Cat	Cui, et al
34	ON893141	SYMN003	Bat	Kamau J,et al
35	OR572101	Y4a	Bat	This study
36	OR761825	Y4b	Bat	This study
37	OR761826	Y6a	Bat	This study
38	OR761827	Y6b	Bat	This study
39	OR761828	Y7b	Bat	This study

* Cui *et al.*, 2012b. Discovery of retroviral homologs in bats: implications for the origin of mammalian gammaretroviruses.

Journal of Virology 86(8), 4288-4293.

*Kamau J, *et al.*, 2022. A novel coronavirus and a broad range of viruses in Kenyan cave bats. Viruses 14:2820.
